# Supplementary material for: Upregulation of the JAK-STAT pathway promotes maturation of human embryonic stem cell-derived cardiomyocytes
Source: Stem Cell Reports. 2021 Nov 11;16(12):2928–41. doi: 10.1016/j.stemcr.2021.10.009 (PMC8693666; doi:10.1016/j.stemcr.2021.10.009)
Supplement: Document S1. Figures S1–S7 and Tables S1–S5 [file mmc1.pdf]

Stem Cell Reports, Volume *16*

Supplemental Information

Upregulation of the JAK-STAT pathway promotes maturation of human embryonic stem cell-derived cardiomyocytes

Beatrice Xuan Ho, Hongbing Yu, Jeremy Kah Sheng Pang, Jin-Hui Hor, Lee Chuen Liew, Piotr Szyniarowski, Christina Ying Yan Lim, Omer An, Henry He Yang, Colin L. Stewart, Woon Khiong Chan, Shi-Yan Ng, and Boon-Seng Soh

Up-regulation of JAK-STAT pathway promotes maturation of human embryonic stem cell-derived cardiomyocytes

Beatrice Xuan Ho[1,2,*], Hongbing Yu[1,*], Jeremy Kah Sheng Pang[1,2] , Jin-Hui Hor[1,2,3], Lee Chuen Liew[1] , Piotr Szyniarowski[4], Christina Ying Yan Lim[1], Omer An[5], Henry He Yang[5], Colin L. Stewart[4], Woon Khiong Chan[2], Shi Yan Ng[3,6,7,#] and Boon Seng Soh[1,2,#]

[1] Disease Modeling and Therapeutics Laboratory, A*STAR Institute of Molecular and Cell Biology, 61 Biopolis Drive Proteos, Singapore 138673, Singapore.

[2] Department of Biological Sciences, National University of Singapore, 117543, Singapore.

[3] Neurotherapeutics Laboratory, A*STAR Institute of Molecular and Cell Biology, 61 Biopolis Drive Proteos, Singapore 138673, Singapore.

[4] Institute of Medical Biology, 8A Biomedical Grove, Singapore 138648, Singapore

[5] Cancer Science Institute of Singapore, National University of Singapore, Singapore 117599, Singapore

[6] Department of Physiology, National University of Singapore, 2 Medical Dr, Singapore 117593, Singapore.

[7] National Neuroscience Institute, Singapore 308433

* Equal contribution

# Correspondence: **Shi-Yan Ng, Ph.D**

A*STAR Institute of Molecular and Cell Biology,

61 Biopolis Drive, Proteos, Singapore 138673.

Tel: (65) 6586 9525

Fax: (65) 6779 1117

Email: syng@imcb.a-star.edu.sg

Boon-Seng Soh, Ph.D

A*STAR Institute of Molecular and Cell Biology,

61 Biopolis Drive, Proteos, Singapore 138673.

Tel: (65) 6586 9530

Fax: (65) 6779 1117

Email: bssoh@imcb.a-star.edu.sg

Supplementary Information

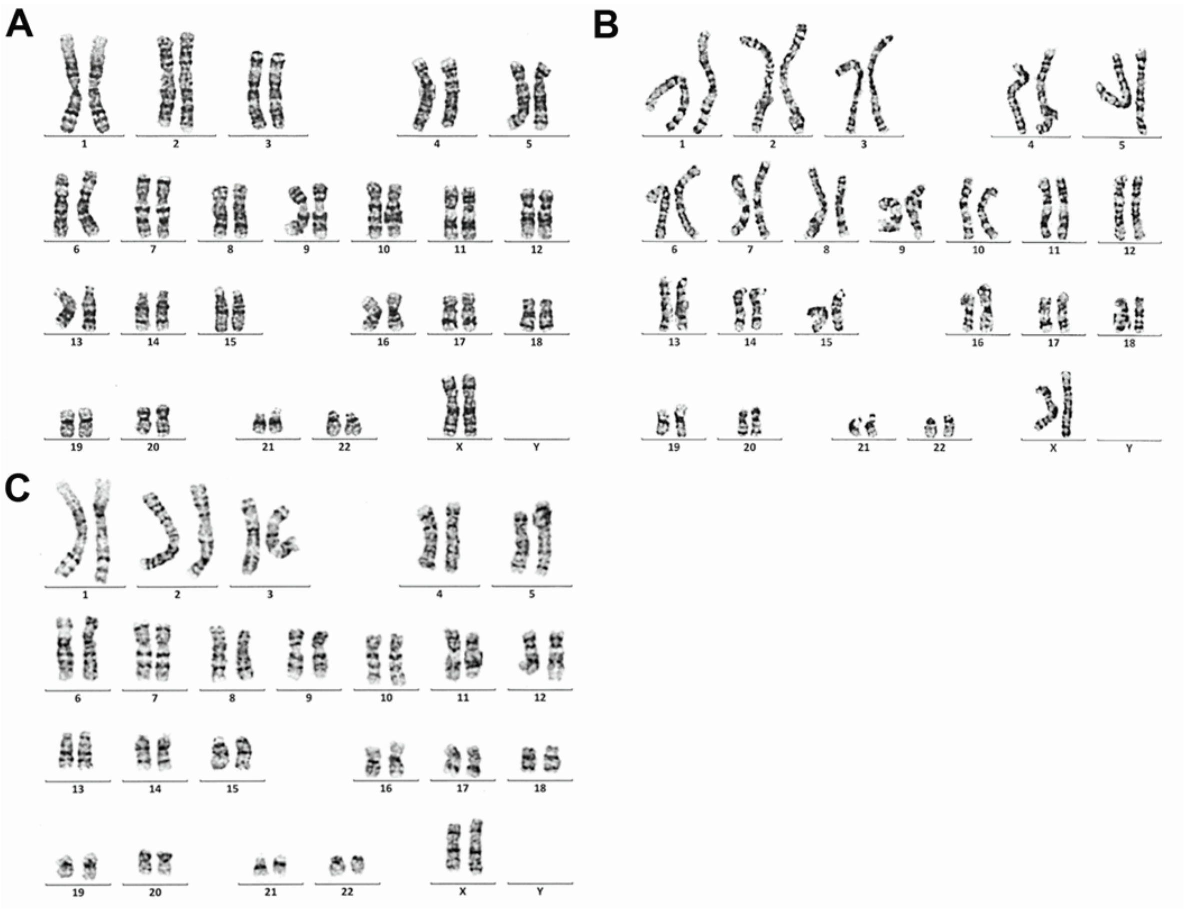

Figure S1. Karyotype of H7 HK1-GFP cell line.
(A-C) Normal female karyotype of human embryonic stem cell line, H7-HK1-GFP clones 1, 2 and 3 were used in this study.

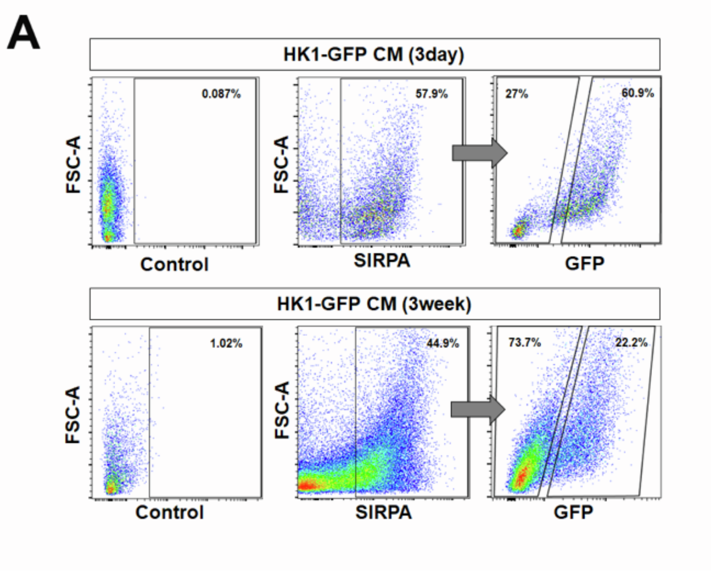

Figure S2. Representative flow cytometry analysis of H7 HK1-GFP derived CMs stained with SIRPA and sorted on 3-day and 3-week post-initial contraction.
(A) H7 HK1 derived CM, 3-day post initial contraction population showed 57.9% of the cells were SIRPA+, with 27% low-GFP and 60.9% high-GFP expressing CM. While H7 HK1 derived CM, 3-week post initial contraction population showed 44.9% of the cells were SIRPA+, with 73.7% low-GFP and 22.2% high-GFP expressing CM.

A

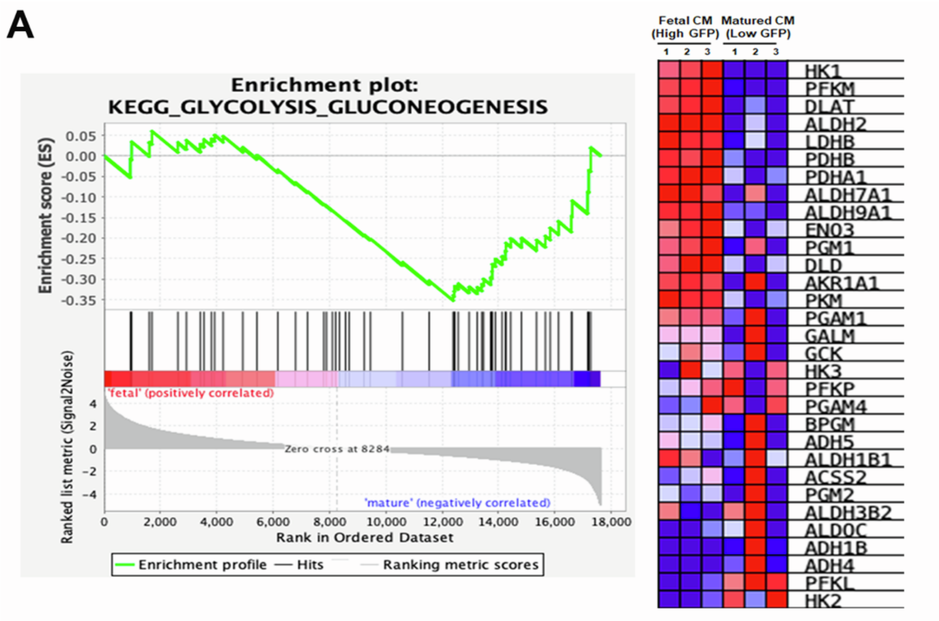

Figure S3. Heatmap showing relative expression of glycolysis and gluconeogenesis related genes.
(A) Gene set enrichment analysis (GSEA) showing downregulation of glycolysis and gluconeogenesis related genes in 3-week post initial contracting CM (expressing low GFP) compared to 3-day post initial contracting CM (expressing high GFP).

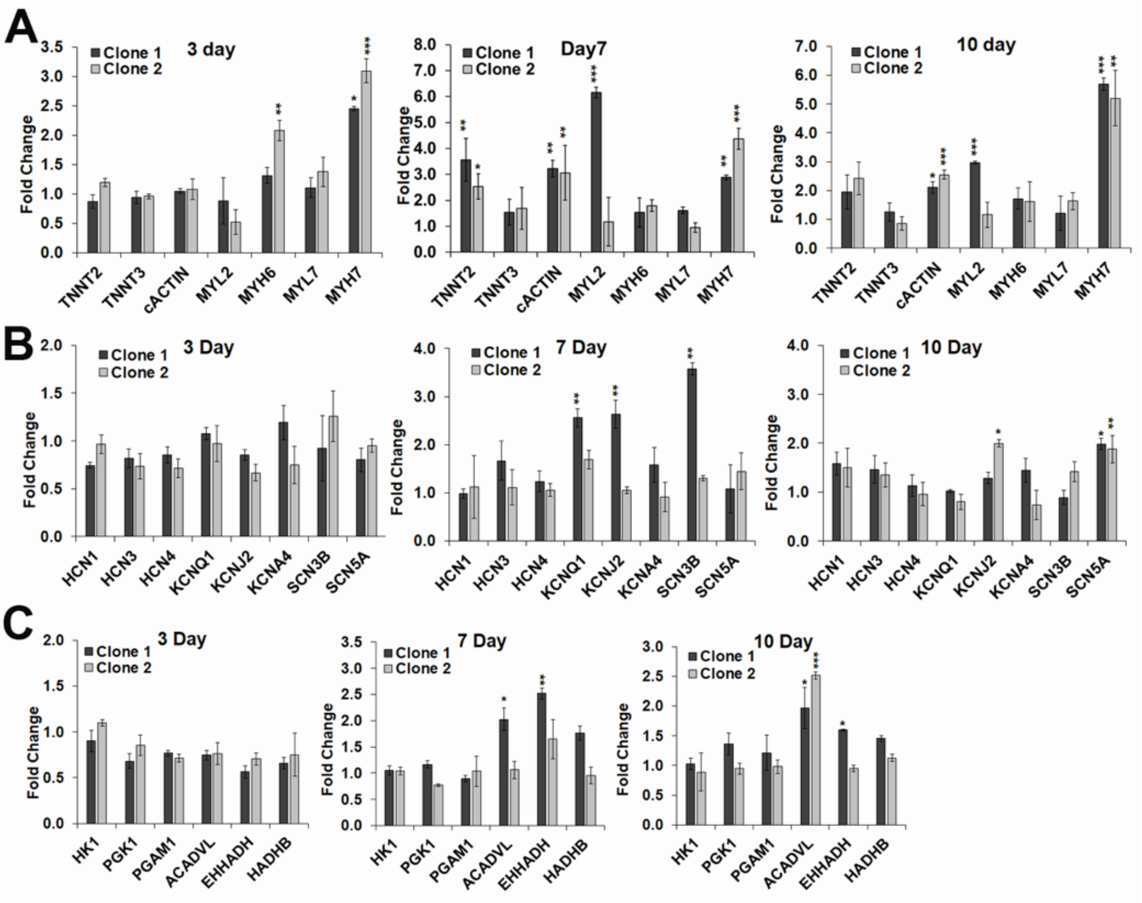

Figure S4. Relative mRNA expression levels of sarcomere, ion channel and metabolic genes in ES03-derived cardiomyocytes.

(A) Quantitative-PCR analysis illustrated increased mRNA transcript expression of sarcomere,

(B) ion channel, and

(C) metabolic genes, in IFN-γ-treated ES03-derived CM (clones 1 and 2), as compared to control. Data is represented as fold-change normalized to control samples, while expression of each gene was normalised to *β-ACTIN*. Data are presented as mean ± SD, n = 3 batches of independently differentiated groups. Data information: Statistical analysis was performed using students two-tailed T-test. * $P < 0.05$, ** $P < 0.01$, *** $P < 0.001$.

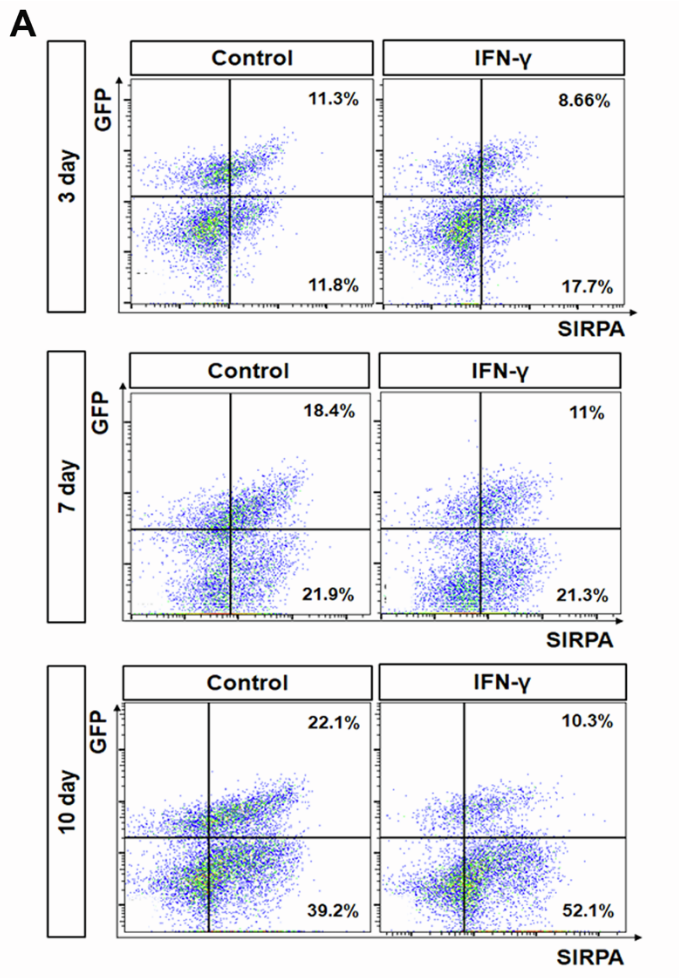

Figure S5. Flow cytometry analysis of relative HK1-GFP expression in H7-HK1 derived cardiomyocytes treated with IFN-γ.
(A) Representative flow cytometric analysis showing percentage of low:high GFP expressing cardiomyocytes in 3 day, 7 day and 10 day post IFN-γ-treated H7-derived CMs compared to control. n = 3 independently differentiated groups.

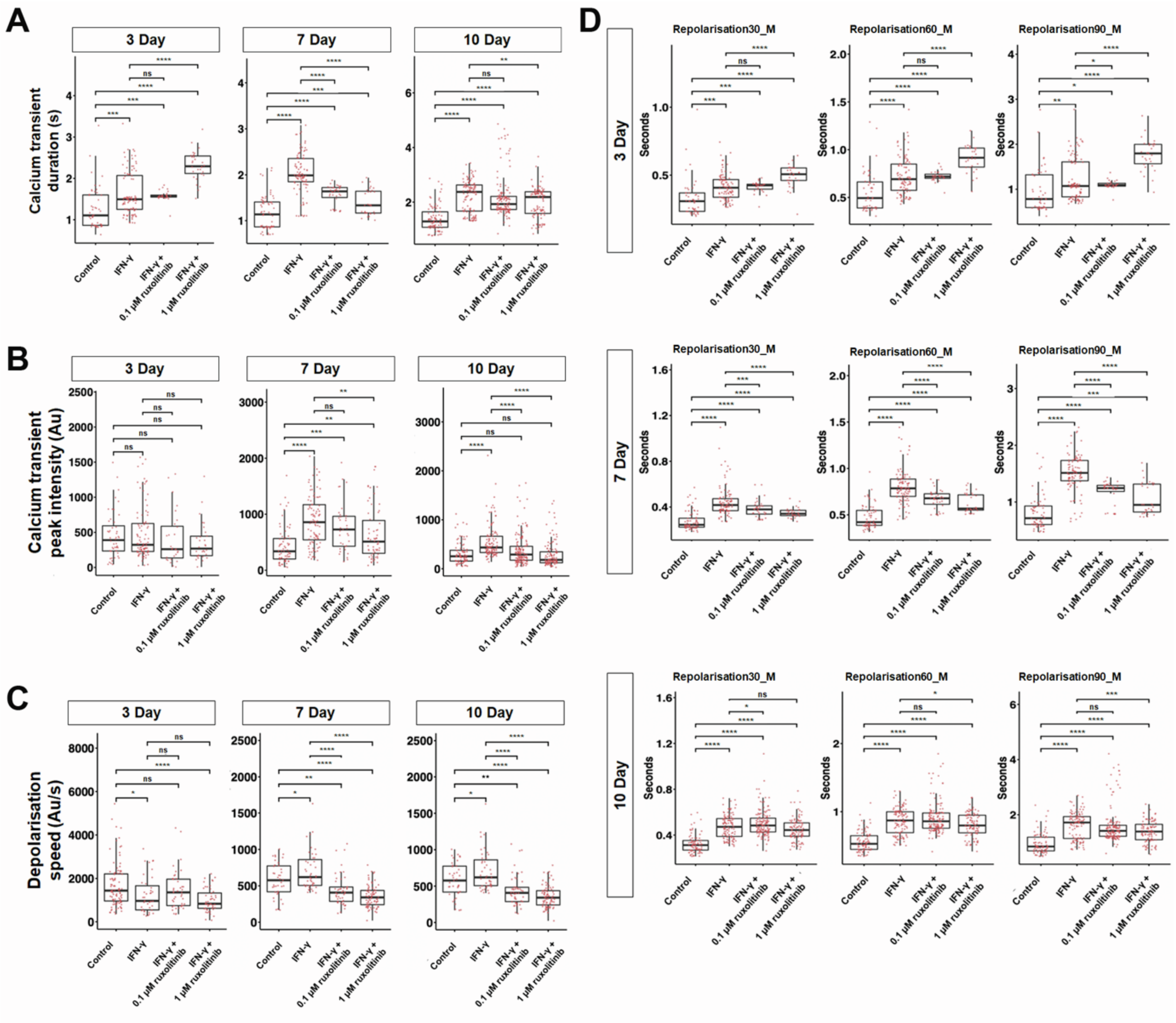

Figure S6. IFN-γ treated ES03-GCaMP6s expressing cardiomyocytes exhibit functional maturation.
(A-D) Representative data of one of the two ES03-GCaMP6s clone derived CMs that were differentiated and analysed.
(A) Dot plot showing mean calcium transient duration (s),
(B) calcium transient peak intensity (Au),
(C) depolarisation speed (Au/s), and
(D) repolarisation duration (30th, 60th, 90th percent) per cell, measured across 30 seconds. n = 50 cells, 3 independently differentiated groups, 2 clones per cell line (ES03). Data information: Statistical analysis was performed using students two-tailed T-test. * P < 0.05, **P < 0.01, ***P < 0.001, ****P < 0.0001.

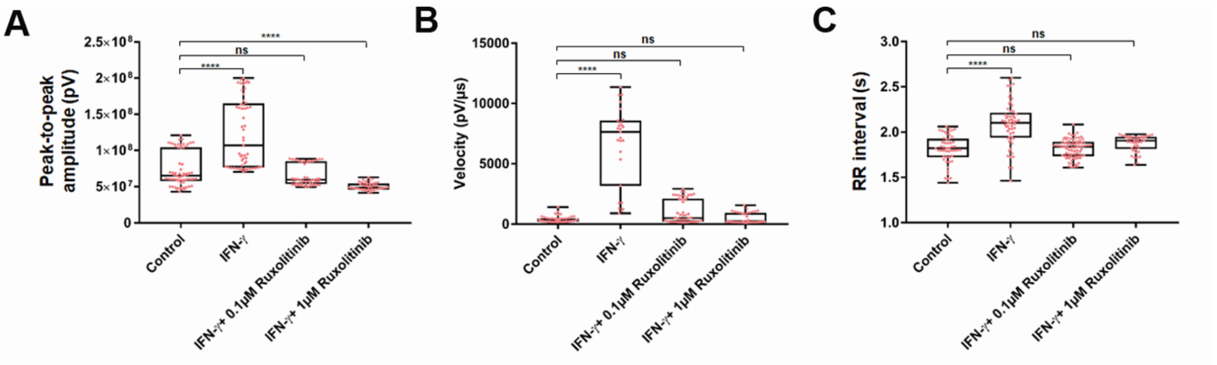

Figure S7. Electrophysiological assessment of IFN-γ-treated G608G human iPS-derived fetal cardiomyocytes using high-throughput multielectrode array (MEA) system.
(A) Dot plot showing mean peak-to-peak amplitude (pV),
(B) velocity (pV/ms), and
(C) RR interval of G608G human iPS-derived fetal CMs (10 days post IFN-γ treatment) measured across 5 min. n = 3 independently differentiated groups. *P < 0.05, **P < 0.01, ***P < 0.001, ****P < 0.0001.

SUPPLEMENTAL MULTIMEDIA FILES

Video S1
Representative video of spontaneously contracting ES03-derived cardiomyocytes cultured in RPMI/B27.

Video S2
Representative video of spontaneously contracting ES03-derived cardiomyocytes cultured in RPMI/B27 supplemented with 25ng/ml IFN-γ.

SUPPLEMENTAL TABLES

Table S1
PCR primers used for genotyping of HK1-GFP report cell line.

**GT-LH-F**	CCATGTCAGCCCCTAACATCC
**GT-LH-R**	CGACGTCACCGCATGTTAGC
**GT-RH-F**	GCATCGCATTGTCTGAGTAGG
**GT-RH-R**	CCAGCCATTAAGCATCACTCG

Table S2
List of Quantitative-PCR primers used in this study.

Gene	Forward primer (5' – 3')	Reverse primer (5' – 3')
MEF2C	ATGGATGAACGTAACAGACAGGT	CGGCTCGTTGTACTCCGTG
NKX2.5	CCAAGGACCCTAGAGCCGAA	ATAGGCGGGGTAGGCGTTAT
TBX5	TTGCATGTATGCCAGCTCTG	CTGGTAGGGTAGCCTGTCC
cACTIN	TCCTGATGCGCATTTTTATTC	AACACCACTGCTCTAGCCACG
TNNT2	CAGAGCGGAAAAGTGGGAAGA	TCGTTGATCCTGTTTCGGAGA
TNNT3	GCCCACCTCAAGCAGGTG	TTGCGCCAGTCTCCCACCTC
HCN1	TGAAGCTGACAGATGGCTCTT	CTGGCAGTACGACGTCCTTT
HCN2	CTGATCCGCTACATCCATCA	AGATTGCAGATCCTCATCACC
HCN4	TCGACTCGGAGGTCTACAAG	GGTCGTAGGTCATGTGGAAG
KCNQ1	ATGGTGCGCATCAAGGAG	GATGAACAGTGAGGGCTTCC
KCNJ2	TGGCCAGGCTCATGTGTAG	CAAAAGGAAAAGCCCAGAAA
SCN3B	CATTCTGTAGCCCAGACGGG	GGGTAAGCTCAGCTCGGAAG
SCN5A	CACGCGTTCACTTTCCTTC	AAGAGCCGACAAATTGCCTA
CACNA1G	GAAGCTGATGGACGAGCTG	CCATCTCAGCTAGAGGGATCTG
MYL2	GCGGAGTGTGGAATTCTTCT	GTCAATGAAGCCATCCCTGT
HK1	TTCACGGAGCTGAAGGATGA	CTTCCTGAAGCGAGTCATGA
PGK1	GAATCACCGACCTCTCTCCC	GGGACAGCAGCCTTAATCCT
PGAM1	GAGCCCGACCATCCTTTCTA	CAGTACACGTTTCCCCTCCT
PPARA	GCTATCATTACGGAGTCCACG	TCGCACTTGTCATACACCAG
PPARG	GAGCCCAAGTTTGAGTTTGC	GCAGGTTGTCTTGAATGTCTTC
ACADVL	ATGGTGGTGGTTCTCTCGAG	GGCCTTGGAGATGCTTTTGA
EHHADH	GCATCGTGGAAAACAGCATC	CCGAGTCTACAGCAATCACAG
HADHB	ACCATGGCTTGTATCTCTGC	CTTGAGTGACGAATAGGGACATC
STAT1	ATGGCAGTCTGGCGGCTGAATT	CCAAACCAGGCTGGCACAATTG
IRF1	AAAGTCGAAGTCCAGCCGAG	TGTTGTAGCTGGAGTCAGGG
IRF2	AGGACAGTCCCATCTGGACA	ATTCCTCTTCCGCCAGTGTG
ISG20	ACACGTCCACTGACAGGCTGTT	ATCTTCCACCGAGCTGTGTCCA

β-ACTIN	CCAACCGCGAGAAGATGA	CCAGAGGCGTACAGGGATAG

Table S3
List of differentially expressed transcription factors of top 8 pathways between 3-week (relatively more matured CM expressing low GFP) and 3-day (fetal-like CM expressing high GFP) post initial contracting CMs (log2 fold change). Arranged in descending order from left (top to bottom), followed by right (top to bottom).

Up-regulated transcription factors in matured low-GFP expressing CM	Down-regulated transcription factors in matured low-GFP expressing CM
**Gene**	**Gene**
SP100	*TOP3A*
IRF8	*NUP153*
ATF3	*PIAS4*
IRF5	*CSNK1E*
IRF4	*PSMD10*
DDIT3	*CSNK2A2*
IRF1	*PRKACA*
IRF6	*NEK9*
ISG20	*FBXO5*
FRK	*VRK1*
CREB3L3	*MAPK3*
IRF9	*MDC1*
DNAJB9	*CHEK1*
EIF2AK3	*BRCA1*
STAT1	*CDC7*
NFKB1	*PLK4*
XBP1	*HERC2*
DNAJB11	*BUB1*
DNAJC3	*NEK2*
RNASEL	*PLK1*
ERN1	*PKMYT1*
IRF2	*AURKB*
PRKCD	*CDK1*
ATF4	*PRKCB*

Table S4
List of up-regulated genes 3-week (relatively more matured CM expressing low GFP) and 3-day (fetal-like CM expressing high GFP) post initial contracting CMs. All the genes were found to be associated with respective pathways with entities FDR less than 0.05 and P-value less than 0.01 listed.

Pathway name	Gene		P-value
**Neutrophil Degranulation**	*NEU1*	*LAMTOR3*	2.31E-08
	AHSG	*SERPINB6*	
	C3	*CD63*	
	HSPA6	*ORM2*	
	ANXA2	*DDX3X*	
	CTSH	*ITGAM*	
	FTL	*LAMTOR3*	
	SERPINA3	*SERPINB6*	
	CEACAM1	*SLPI*	
	GLIPR1	*HSPA1B*	
	LGALS3	*DNAJC3*	
	CTSS	*SLC2A5*	
	CD300A	*HSP90AA1*	
	FUCA1	*PDXK*	
	CXCL1	*LYZ*	
	AGPAT2	*OLFM4*	
	ORM1	*PRSS3*	
	HPSE	*RNASET2*	
	LRRC7	*ACAA1*	
	TLR2	*NFASC*	
	LPCAT1	*ASAH1*	
	SNAP25	*PRDX4*	
	QSOX1	*ABCA13*	
	ERP44	*C3AR1*	
	CD59	*CTSA*	
	DPP7	*STK11IP*	

	STBD1	*CD44*	
	MANBA	*AMPD3*	
	HEXB	*VNN1*	
	SLC15A4	*IDH1*	
	BRI3	*CD58*	
	CEACAM6	*ORMDL3*	
	ITGAL	*ARL8A*	
	AGA	*SERPINB1*	
	FUGA2	*TOLLIP*	
	LAMP2	*CD55*	
	FAF2	*ITGAX*	
	SVIP	*PYCARD*	
	TMEM173	*B2M*	
	ADAM8	*RAB18*	
	OLR1	*CD47*	
	RAB24	*PTAFR*	
	FCGR2A	*QPCT*	
	CYSTM1	*FTH1*	
	GSN	*ALDH3B1*	
	LRG1	*ALAD*	
	TMBIM1	*CYBA*	
	P2RX1	*A1BG*	
	PLD1	*NIT2*	
	HSPA1A	*ARHGAP45*	
	LRMP	*CTSZ*	
	FRK	*COPB1*	
	MAGT1	*HLA-C*	
	S100P	*RAP1A*	
	NBEAL2	*DSC1*	

	CHI3L1	*ANO6*	
	CD68	*VAMP8*	
	NFKB1	*ALOX5*	
	CD63	*HLA-B*	
	ORM2	*CPPED1*	
	DDX3X	*ITGAV*	
	ITGAM	*PRKCD*	
	SNAP23	*MMP9*	
	NHLRC3	*TNFAIP6*	
**Interferon alpha/beta signaling**	*IRF1*	*MX2*	7.19E-08
	GBP2	*IFNAR1*	
	PSMB8	*XAF1*	
	MX1	*IFNAR2*	
	STAT1	*IRF9*	
	IRF5	*IRF6*	
	ISG20	*HLA-F*	
	IFIT2	*RNASEL*	
	IRF2	*OAS3*	
	IRF8	*IP6K2*	
	OAS1	*HLA-C*	
	IFIT3	*TYK2*	
	JAK1	*RSAD2*	
	HLA-G	*HLA-A*	
	IRF4	*HLA-B*	
	ATF3	*EIF2AK3*	
	HERPUD1	*ASNS*	
	DDIT3	*TSPYL2*	
	CREBRF	*CREB3L3*	
	CXCL8	*SEC31A*	

**Unfolded protein response (UPR)**	*XBP1*	*KDELR3*	9.90E-08
	SRPRB	*DNAJC3*	
	CXXC1	*HSPA5*	
	DNAJB9	*SSR1*	
	CCL2	*SRPRA*	
	IGFBP1	*ACADVL*	
	EDEM1	*DNAJB11*	
	PDIA5	*HSP90B1*	
	LMNA	*PPP2R5B*	
	WFS1	*MYDGF*	
	ATF4	*YIF1A*	
	GFPT1	*WIPI1*	
	SERP1	*ERN1*	
**Interferon gamma signaling**	*GBP1*	*HLA-G*	9.90E-08
	IRF1	*IRF4*	
	TRIM21	*PTPN2*	
	GBP2	*GBP7*	
	SP100	*IRF9*	
	STAT1	*IRF6*	
	IRF5	*HLA-F*	
	MT2A	*IFI30*	
	TRIM22	*CD44*	
	IRF2	*B2M*	
	IRF8	*OAS3*	
	ICAM1	*PTAFR*	
	GBP3	*HLA-C*	
	TRIM25	*HLA-A*	
	TRIM26	*HLA-B*	
	OAS1	*PRKCD*	

	JAK1	*TRIM29*	
	TRIM31	*IFNGR2*	

Table S5
List of down-regulated genes in 3-week (relatively more matured CM expressing low GFP) and 3-day (fetal-like CM expressing high GFP) post initial contracting CMs. All the genes were found to be associated with respective pathways with entities FDR less than 0.05 and P-value less than 0.01 listed.

Pathway name	Gene		P-value
Mitotic Prometaphase	*DYNC1I1*	*CENPN*	4.57E-17
	SMC1A	*CENPF*	
	ZWILCH	*CENPE*	
	ZWINT	*PRKAR2B*	
	TUBA1B	*BUB1*	
	SPC25	*KIF2C*	
	ERCC6L	*CLASP2*	
	CDCA5	*NDE1*	
	DYNLL2	*HAUS5*	
	CSNK2A2	*CENPL*	
	NUP107	*NDC80*	
	NCAPG	*AURKB*	
	CENPO	*HAUS1*	
	MAD1L1	*NCAPD2*	
	CENPU	*PLK4*	
	BUB1B	*CENPH*	
	SMC4	*SMC2*	
	CENPI	*MAPRE1*	
	PLK1	*NINL*	
	KNL1	*NUF2*	
	SGO1	*MZT1*	
	CEP72	*CDC20*	
	CENPK	*MAD2L1*	
	DCTN1	*NEK2*	
	INCENP	*NUMA1*	

	PPP2R5D	*NEDD1*	
	SGO2	*KNTC1*	
	BIRC5	*PRKACA*	
	SKA1	*PPP1CC*	
	TUBB	*CENPA*	
	SPDL1	*SPC24*	
	YWHAE	*HAUS4*	
	CKAP5	*CCNB2*	
	NCAPH	*CCNB1*	
	XPO1	*CSNK1E*	
	CENPM	*ALMS1*	
	CDCA8	*CDK1*	
**Cell Cycle checkpoints**	*HERC2*	*SGO2*	**1.40E-16**
	DYNC1I1	*BIRC5*	
	MDC1	*SKA1*	
	PKMYT1	*CCNE1*	
	ZWILCH	*SPDL1*	
	ZWINT	*YWHAE*	
	CLSPN	*CKAP5*	
	RMI2	*XPO1*	
	MCM5	*CENPM*	
	MCM2	*CDCA8*	
	SPC25	*CDK1*	
	TP53BP1	*DNA2*	
	ERCC6L	*CENPN*	
	CDC45	*CENPF*	
	MCM4	*CENPE*	
	MCM6	*BUB1*	
	EXO1	*KIF2C*	

	CDC25A	*CLASP2*	
	CDC7	*NDE1*	
	MCM3	*PSMD10*	
	DYNLL2	*CENPL*	
	MCM8	*NDC80*	
	GTSE1	*AURKB*	
	MCM10	*UBE2C*	
	BRCA1	*CCNA2*	
	H2AFX	*CENPH*	
	CDC6	*MCM7*	
	RFC3	*TOP3A*	
	CDKN1B	*PIAS4*	
	NUP107	*ATRIP*	
	CENPO	*MAPRE1*	
	CHEK1	*NUF2*	
	MAD1L1	*CDC20*	
	CENPU	*MAD2L1*	
	BUB1B	*RMI1*	
	CENPI	*KNTC1*	
	PLK1	*RPA2*	
	KNL1	*PSMB1*	
	SGO1	*RFC4*	
	PHF20	*RFC2*	
	CENPK	*ANAPC1*	
	BLM	*PPP1CC*	
	CDC23	*CENPA*	
	INCENP	*ORC6*	
	PPP2R5D	*SPC24*	
	PSME3	*CDC25C*	

	CCNB1	*CCNB2*	
**Amplification of signal from the kinetochores**	*DYNC1I1*	*BIRC5*	**2.67E-15**
	ZWILCH	*SKA1*	
	ZWINT	*SPDL1*	
	SPC25	*CKAP5*	
	ERCC6L	*XPO1*	
	DYNLL2	*CENPM*	
	NUP107	*CDCA8*	
	CENPO	*CENPN*	
	MAD1L1	*CENPF*	
	CENPU	*CENPE*	
	BUB1B	*BUB1*	
	CENPI	*KIF2C*	
	PLK1	*CLASP2*	
	KNL1	*NDE1*	
	SGO1	*CENPL*	
	CENPK	*NDC80*	
	INCENP	*AURKB*	
	PPP2R5D	*CENPH*	
	SGO2	*MAPRE1*	
	NUF2	*KNTC1*	
	CDC20	*PPP1CC*	
	MAD2L1	*CENPA*	
	SPC24		
	DYNC1I1	*SKA1*	
	ZWILCH	*SPDL1*	
	ZWINT	*CKAP5*	
	SPC25	*XPO1*	
	ERCC6L	*CENPM*	

**Mitotic Spindle Checkpoint**	*DYNLL2*	*CDCA8*	**2.39E-14**
	NUP107	*CENPN*	
	CENPO	*CENPF*	
	MAD1L1	*CENPE*	
	CENPU	*BUB1*	
	BUB1B	*KIF2C*	
	CENPI	*CLASP2*	
	PLK1	*NDE1*	
	KNL1	*CENPL*	
	SGO1	*NDC80*	
	CENPK	*AURKB*	
	CDC23	*UBE2C*	
	INCENP	*CENPH*	
	PPP2R5D	*MAPRE1*	
	SGO2	*NUF2*	
	BIRC5	*CDC20*	
	MAD2L1	*PPP1CC*	
	KNTC1	*CENPA*	
	ANAPC1	*SPC24*	

SUPPLEMENTAL EXPERIMENTAL PROCEDURES

Generation of clonal H7 HK1-EGFP reporter cell line

sgRNA sequences were identified using the online CRISPR design tool (crispr.mit.edu). In brief, 100 base pair DNA sequences of the human HK1 gene flanking the stop codon was used for designing the sgRNAs. Two pairs of sgRNAs were selected for cloning into PX459 Cas9 vector. For construction of the HK1 donor vector, V5-2A-EGFP sequence was inserted into pUC19 vector. The HK1 left homologue arm without stop code was inserted upstream of the V5-2A-EGFP sequence, while the HK1 right homologue arm was inserted downstream of the gene. Both left and right homologue arms were validated through sequencing. 10ug HK1 sgRNA and 10ug HK1 donor vector were electroporated into $1x10^6$ the human H7 hES cell line (Thermos-fisher Neon 10ul kit, 1400 V, 30 sec). After 1 week, the electroporated human ES cells were sorted twice for EGFP expression. Single H7 HK1-EGFP positive cells were seeded into 96-well plate for clonal expansion.

PCR validation of HK1 reporter clones

Briefly, GoTaq® Flexi DNA Polymerase (Promega, U.S.A., M829) kit was used in accordance to manufacturer's recommendations. The PCR primers used for genotyping as shown in Table S1.

hESC-derived cardiomyocyte differentiation, sample collection for RNA sequencing and IFN-γ treatment

Upon 80-90% confluence, H7, ES03 and G608G hES/iPS cells were differentiated into CMs via Wnt signalling modulation method as described by Lian *et al*., 2013 (Lian et al. 2013). CMs were FAC sorted after 3-days post intial contraction (expressing high HK1-GFP) and 3-weeks post intial contraction (expressing low HK1-GFP) for RNA sequencing (Refer to Figure 2A).

Prior to IFN-γ treatment, hES-derived CMs were dissociated with accutase and purified with human PSC-derived cardiomyocyte isolation kit (Miltenyi Biotec, Germany) as described on manufacturer's instructions. Enriched cardiomyocyte population was seeded using RPMI/B27 with 5 µM of Y27632 (Miltenyi Biotec, Germany) onto on culture plates coated with Matrigel® matrix (Corning, U.S.A.) diluted in DMEM/F12 (Gibco, U.S.A.). On day 10, contracting CMs were treated with 25ng/ml IFN-γ (Miltenyi Biotec; 130-096-482) and ruxolitinib (Selleckchem; NCB018424) in RPMI/B27 for 3 days, 7 days and 10 days, as illustrated in figure 4A.

SDS-PAGE and western blot

Cells were lysed with RIPA buffer (ThermoScientific, U.S.A.) with the addition of cOmplete™ Protease

Inhibitor Cocktail (Sigma Aldrich, U.S.A.). Cells that require preservation of phosphorylation state were lysed with PhosphoSafe™ Extraction Reagent (Novagen; #71296-3). Protein extracts were then quantified using Pierce™ BCA Protein Assay Kit (Thermo Scientific, U.S.A.) in accordance to manufacturer's recommendations. Protein lysates were resolved in 8-12% SDS-PAGE gels in Tris-Glycine-SDS buffer, transferred to a PDVF membrane (Bio-Rad, U.S.A.) using the Trans-Blot® Turbo™ transfer system (Bio-Rad, U.S.A.) or conventional wet transfer methods. Primary antibodies used include mouse anti-V5 antibody (ThermoScientific, U.S.A.), mouse anti-HK1 antibody (ThermoScientific, U.S.A.), mouse anti α-tubulin (Cell signaling technologies, #2148S), mouse anti-β-actin (Cell signaling technologies, 8H10D10), rabbit anti-GAPDH (Cell signaling technologies, D16H11), rabbit anti-STAT1 (Cell signaling technologies, D1K9Y), rabbit anti-phospho STAT1 (Cell signaling technologies, Y701), rabbit anti-MYH7 (Abcam, ab172967) and anti-MYH7 (Santa cruz, sc-53090) were prepared according to manufacturer's instructions. Secondary antibodies conjugated with horseradish peroxidase (Santa Cruz Biotechnology, U.S.A.) were used. Membranes were then exposed to Clarity™ ECL Western Substrate (Bio-rad, U.S.A.).

Fluorescence-activated cell sorting and flow cytometric analysis

Cardiomyocyte were first dissociated using StemPro-Accutase®(Gibco, U.S.A.) and stained with SIRPA antibody conjugated with SIRPA-conjugated PE-Cy7 (NEB) (1:250 dilution) in blocking buffer (1% FBS in PBS) for 2 h at 37 °C and washed thoroughly with PBS before the cells were resuspended in fluorescence-activated cell sorting (FACs) buffer (0.5% FBS and 1% BSA in PBS). FACS was performed using the LSR II (BD Biosciences, U.S.A.).

For intracellular staining, cells were fixed in 4% PFA (Nacalai Tesque, Japan) at room temperature for 10 min, before incubation in blocking buffer (PBS with 1% Fetal Bovine Serum; Gibco, U.S.A.) with 0.1% Triton-X (Promega, U.S.A.) for 10 min, followed by staining in blocking buffer with rabbit anti-MYH7 antibody (1:250, ab172967; Abcam) for 2 h at room temperature. The cells were washed with PBS and centrifuged at 1200rpm for 5min, the cells were stained in Alexa Fluor® 594 (1:1000) (Invitrogen, U.S.A.) for 1-1.5 h at room temperature in the dark. The cells were washed thoroughly with PBS before resuspension in FACS buffer (0.5% FBS, 1%BSA in PBS). Flow cytometry analysis was performed with LSR II (BD Biosciences, U.S.A.).

RNA Extraction and RT-qPCR

CMs were lysed in 300 µl of TRIzol reagent (Invitrogen, U.S.A.) followed by RNA extraction and precipitation from the aqueous phase and removal of genomic DNA. RNA samples (250-500 ng) were reverse transcribed using High-Capacity cDNA Reverse Transcription Kit (Applied Biosystems, U.S.A.). qPCR was performed using the FAST SYBR Green Mix (Applied Biosystems, U.S.A.), 0.5 µM of specific primers (Table S2) and ~5 ng of cDNA. $\Delta\Delta C_T$-based relative quantification method was adopted for qPCR analysis using the QuantStudio 5 384-well Block Real-Time PCR system (Applied Biosystems, U.S.A.). Data is presented as fold-change where CT values were normalised to *β-ACTIN*. Data presented are representative of three independent experiments with error bars indicative of the standard deviation (SD) unless otherwise stated.

Metabolic flux analysis using Seahorse XFe96 Analyzer

Extracellular acidification rates (ECAR) were measured using a XFe96 Seahorse Biosciences Extracellular Flux Analyzer (Agilent Technologies). Purified CMs were plated onto a Matrigel pre-coated Seahorse 96-well plate at 125,000 CMs per well 24 hours prior to the assay. Culture media were changed with 175ul of fresh Seahorse DMEM basal medium 45 minutes prior to the assay. Seahorse analyzer injection ports were filled with 10mM glucose, 1µM oligomycin and 50mM 2-DG, following the manufacturer's instructions. Levels of ECAR were recorded, normalized and quantified based on manufacturer's instructions.

Immunostaining, confocal microscopy and image analysis

Cells were then fixed using 4% PFA (Nacalai-Tesque, Japan) for 10 min at room temperature, followed by permeabilisation with 0.2% Triton-X (Promega, U.S.A.) for 10 min. The cells were then treated with blocking buffer for 30 min. Primary antibody anti-sarcomeric α-actinin (1:150, ab9465, Abcam) was incubated at 4 °C overnight, followed by incubation with the appropriate Alexa Fluor® 488 and 594 fluorescent dyes for 1.5 hours, and counter-stained with 4,6-diamindino-2-phenylindole (DAPI) (AAT Biorequest, U.S.A.). For this study, primary antibody used were mouse α-actinin (1:150, ab9465, Abcam). Imaging was performed using the Olympus Fluoview inverted confocal microscope (Olympus, U.S.A.) using oil immersion for 100x optical zoom. Sarcomere length and myofibril width were measured with freehand drawing tools in Fiji based on α-actinin staining. Sarcomere length and myofibril width were measured automatically using a custom Fiji plug-in, MyofibrilJ, available from (https://imagej.net/MyofibrilJ). All measurements were based on α-actinin staining. Full details of analysis was performed as mentioned by Spletter *et al.,* (Spletter et al. 2018). Data were recorded in Excel and statistical analysis comparing two groups were performed by mean of a two-tailed

unpaired Student's t-test. P values lower than 0.05 were considered significant. A computational tool, ZlineDetection was performed on MATLAB to determine skewness of continuous z-line (CZL) and z-line fraction as described by *Morris et al* (Morris et al. 2020). All measurements were based on α-actinin (green) and phalloidin (red) staining. Statistical analysis comparing two groups were performed by mean of a two-tailed unpaired Student's t-test. P values lower than 0.05 were considered significant.

Mitotracker staining, image acquisition and image analysis

MitoTracker® Red FM, far red-fluorescent dye (abs/em ~581/644 nm) was used to stain mitochondria in live CMs. Images of FAC sorted CMs (low/high-GFP expressing) on 96-well plates were acquired using the high content microscope Operetta (Perkin Elmer) using the 20x objective. Image analyses including intensity measurements were performed using Columbus (Perkin Elmer). Nuclei were detected in live cells with hoescht (blue) stain, with dead cells filtered based on abnormally high hoescht intensity and small ($<20\ \mu m^2$) nuclei area. Intensity of mitotracker staining within cytoplasm was determined and a cut-off above background intensity was used to identify CMs.

Gene expression profiling and pathway enrichment analysis

Gene expression profiling and pathway enrichment analysis have been performed by using CSI NGS Portal (An et al. 2019). Briefly, raw fastq files were trimmed by Trimmomatic (Bolger, Lohse, and Usadel 2014) for adapter removal. The clean reads were aligned to the reference human genome (hg19) by using STAR (Dobin et al. 2013) (v2.7.3a) with default parameters. The gene expression quantification was done by using HTSeq-count(Anders, Pyl, and Huber 2015) (v0.11.2) in strand-specific mode "-s reverse" to obtain raw read counts for each gene, and read counts only from the sense strand are used. First, PCA and hierarchical clustering were performed with regionReport (Collado-Torres, Jaffe, and Leek 2015) to compare the gene expression profiles of the samples, by using the top 500 genes with the highest variance across the samples. Then the differential gene expression analysis was performed by using DESeq2 (Love, Huber, and Anders 2014) (v1.24.0) starting from the raw read counts by comparing the samples from C9 Matured Low GFP to C9 Fetal Hi GFP after collapsing the three samples within each group as replicates. The genes that are not expressed or lowly expressed (read counts <= 2 on average per sample) were removed from the analysis. In total, 3128 and 2630 genes were significantly up- and down-regulated, respectively, in the 3-week post initial contracting CMs, as compared to the 3-day post initial contracting CMs, after correction for multiple hypothesis testing ($P_{adj} < 0.05$, using

Benjamini-Hochberg method). These differentially expressed genes were used for the pathway enrichment analysis (ReactomePA database) (Yu and He 2016). Additionally, gene set enrichment analysis (GSEA(Subramanian et al. 2005)) was performed by using the gene expression data for all the genes normalized by DESeq2 (Love, Huber, and Anders 2014). To be comprehensive, all pathways and gene sets were selected as the input for the enrichment analyses. The significantly altered pathways were identified and plotted as barplot by using R packages ReactomePA (Yu and He 2016) and enrichplot (Yu 2018), respectively.

Fluorescent imaging of intracellular calcium transient

Intracellular calcium kinetics was measured using H7- and ES03-GCaMP6s derived CMs. In brief, the cells were seeded on a 24 well plate pre-coated with matrigel. Prior to imaging, the cells were washed with PBS, and fresh RPMI/B27 medium was added. Spontaneous calcium transients of 3 day, 7 day, 10 day IFN-γ , 0.1μM and 1μM ruxolitinib treated CMs and untreated controls were imaged under a 20x objective Nikon ECLIPSE Ti-S fluorescent microscope and recorded using an Andor Zyla 4.2 sCMOS for up to 1 minute. Calcium transients based on fluorescence intensity over time were computed into contraction peaks in Rstudio. The contraction peaks were computed into the following variables; (1) calcium transient duration (seconds), (2) calcium transient peak intensity (Au), (3) depolarisation speed (mean fluorescence per second), and (4) repolarisation duration (Repolarisation30_M, Repolarisation60_M, Repolarisation90_M) by taking the mean of the contraction intervals respectively. Statistical significance using Student's *t*-test, two sided based on assumed normal distribution.

Multielectrode array (MEA) recording

PEDOT-coated 24-well micro-gold multielectrode array dishes (catalog number 24W300/30G-288, Multi Channel Systems MCS, Germany) contained an array of 12 electrodes per well. Fetal-like CMs (7-10 days) were seeded at a density of 350,000 cells/well that were pre-coated with Matrigel® matrix (Corning, U.S.A.) diluted in DMEM/F12 (Gibco, U.S.A.). CMs were treated with 25ng/ml IFN-γ, 0.1μM and 1μM Ruxolitinib in RPMI/B27 the following day. Field potentials (FP) were recorded 10 days after IFN-γ treatment, at a sampling rate of 20 kHz unless otherwise noted, with a recording duration of 5 min and 2 min wash-in phase. High- and low-pass filter cut-off frequency of 1 Hz and 3500 Hz, respectively were used for data acquisition. A separate software, Multiwell-Analyzer (Multi Channel Systems MCS, Germany) was used to analyse and extract data from the recording. The "RR Internal" measures the time span between two heartbeats, measured from the

detection time stamp of one QRS-component to the next. "Peak-to-Peak amplitude" measures the amplitude between the maximum and the minimum of a Na peak. “Velocity” was measured based on "Peak-to-Peak amplitude" divided by the duration.

Statistical analysis

Quantitative PCR (qPCR) values are expressed as mean ± SD (unless otherwise stated), n = 3 independently differentiated groups. Results were tested for statistical significance using Student’s *t*-test, two sided based on assumed normal distribution. * $P < 0.05$, ** $P < 0.01$, *** $P < 0.001$.

References

An, Ö., K. Tan, Y. Li, J. Li, C. Wu, B. Zhang, L. Chen, and H. Yang. 2019. 'CSI NGS Portal: An Online Platform for Automated NGS Data Analysis and Sharing', *Preprints*, 2019100146.

Anders, S., P. T. Pyl, and W. Huber. 2015. 'HTSeq--a Python framework to work with high-throughput sequencing data', *Bioinformatics*, 31: 166-9.

Bolger, A. M., M. Lohse, and B. Usadel. 2014. 'Trimmomatic: a flexible trimmer for Illumina sequence data', *Bioinformatics*, 30: 2114-20.

Collado-Torres, L., A. E. Jaffe, and J. T. Leek. 2015. 'regionReport: Interactive reports for region-level and feature-level genomic analyses', *F1000Res*, 4: 105.

Dobin, A., C. A. Davis, F. Schlesinger, J. Drenkow, C. Zaleski, S. Jha, P. Batut, M. Chaisson, and T. R. Gingeras. 2013. 'STAR: ultrafast universal RNA-seq aligner', *Bioinformatics*, 29: 15-21.

Lian, X., J. Zhang, S. M. Azarin, K. Zhu, L. B. Hazeltine, X. Bao, C. Hsiao, T. J. Kamp, and S. P. Palecek. 2013. 'Directed cardiomyocyte differentiation from human pluripotent stem cells by modulating Wnt/beta-catenin signaling under fully defined conditions', *Nat Protoc*, 8: 162-75.

Love, M. I., W. Huber, and S. Anders. 2014. 'Moderated estimation of fold change and dispersion for RNA-seq data with DESeq2', *Genome Biol*, 15: 550.

Morris, T. A., J. Naik, K. S. Fibben, X. Kong, T. Kiyono, K. Yokomori, and A. Grosberg. 2020. 'Striated myocyte structural integrity: Automated analysis of sarcomeric z-discs', *PLoS Comput Biol*, 16: e1007676.

Spletter, M. L., C. Barz, A. Yeroslaviz, X. Zhang, S. B. Lemke, A. Bonnard, E. Brunner, G. Cardone, K. Basler, B. H. Habermann, and F. Schnorrer. 2018. 'A transcriptomics resource reveals a transcriptional transition during ordered sarcomere morphogenesis in flight muscle', *Elife*, 7.

Subramanian, A., P. Tamayo, V. K. Mootha, S. Mukherjee, B. L. Ebert, M. A. Gillette, A. Paulovich, S. L. Pomeroy, T. R. Golub, E. S. Lander, and J. P. Mesirov. 2005. 'Gene set enrichment analysis: a knowledge-based approach for interpreting genome-wide expression profiles', *Proc Natl Acad Sci U S A*, 102: 15545-50.

Yu, G., and Q. Y. He. 2016. 'ReactomePA: an R/Bioconductor package for reactome pathway analysis and visualization', *Mol Biosyst*, 12: 477-9.

Yu, Guangchuang. 2018. 'Visualization of Functional Enrichment Result', *R package version 1.2.0*.
