# Supplementary material for: Upregulation of the JAK-STAT pathway promotes maturation of human embryonic stem cell-derived cardiomyocytes
Source: Stem Cell Reports. 2021 Nov 11;16(12):2928–41. doi: 10.1016/j.stemcr.2021.10.009 (PMC8693666; doi:10.1016/j.stemcr.2021.10.009)
Supplement: Document S2. Article plus supplemental information [file mmc4.pdf]

Stem Cell Reports
Article
OPEN ACCESS

Upregulation of the JAK-STAT pathway promotes maturation of human embryonic stem cell-derived cardiomyocytes

Beatrice Xuan Ho,[1,2,8] Hongbing Yu,[1,8] Jeremy Kah Sheng Pang,[1,2] Jin-Hui Hor,[3] Lee Chuen Liew,[1] Piotr Szyniarowski,[4] Christina Ying Yan Lim,[1] Omer An,[5] Henry He Yang,[5] Colin L. Stewart,[4] Woon Khiong Chan,[2] Shi-Yan Ng,[3,6,7,*] and Boon-Seng Soh[1,2,*]
[1]Disease Modeling and Therapeutics Laboratory, A*STAR Institute of Molecular and Cell Biology, 61 Biopolis Drive Proteos, Singapore 138673, Singapore
[2]Department of Biological Sciences, National University of Singapore, Singapore 117543, Singapore
[3]Neurotherapeutics Laboratory, A*STAR Institute of Molecular and Cell Biology, 61 Biopolis Drive Proteos, Singapore 138673, Singapore
[4]A*STAR Skin Research Labs, 8A Biomedical Grove #06-40, Immunos, Singapore 138648
[5]Cancer Science Institute of Singapore, National University of Singapore, Singapore 117599, Singapore
[6]Department of Physiology, National University of Singapore, 2 Medical Dr, Singapore 117593, Singapore
[7]National Neuroscience Institute, Singapore 308433, Singapore
[8]These authors contributed equally
*Correspondence: syng@imcb.a-star.edu.sg (S.-Y.N.), bssoh@imcb.a-star.edu.sg (B.-S.S.)
https://doi.org/10.1016/j.stemcr.2021.10.009

SUMMARY

The immature characteristics and metabolic phenotypes of human pluripotent stem cell-derived cardiomyocytes (hPSC-CMs) restrict their applications for disease modeling, drug discovery, and cell-based therapy. Leveraging on the metabolic shifts from glycolysis to fatty acid oxidation as CMs mature, a human hexokinase1-GFP metabolic reporter cell line (H7 HK1-GFP) was generated to facilitate the isolation of fetal or more matured hPSC-CMs. RNA sequencing of fetal versus more matured CMs uncovered a potential role of interferon-signaling pathway in regulating CM maturation. Indeed, IFN-γ-treated CMs resulted in an upregulation of the JAK-STAT pathway, which was found to be associated with increased expression of CM maturation genes, shift from MYH6 to MYH7 expression, and improved sarcomeric structure. Functionally, IFN-γ-treated CMs exhibited a more matured electrophysiological profile, such as increased calcium dynamics and action potential upstroke velocity, demonstrated through calcium imaging and MEA. Expectedly, the functional improvements were nullified with a JAK-STAT inhibitor, ruxolitinib.

INTRODUCTION

The ability of human pluripotent stem cells (hPSCs) to differentiate into matured cardiomyocytes (CMs) is of paramount importance for drug screening, disease modeling, and cell-based therapy for cardiac regeneration (Funakoshi et al., 2016; Li et al., 2017; Takeda et al., 2018; Yang and Papoian 2018). Although sequential addition of critical growth factors and/or small molecules successfully recapitulates various stages of cardiac specification to a certain extent *in vitro*, the hPSC-CMs generated from these protocols still display fetal-like ultra-structures, electrophysiological properties, and preferential metabolism of glucose, with low expression of key maturation markers (Machiraju and Greenway 2019). Such immaturity not only results in ineffective cardiac contractility but may also lead to arrhythmia (Chen et al. 2009). Hence, enhancing maturation of hPSC-CMs has become the subject of intense research to maximize their potential applications.

In recent years, substantial progress in advancing hPSC-CM maturation has been achieved by utilization of physical and electrical stimulations, biochemical stimulations, microRNA overexpression, metabolic manipulations, as well as 3D engineered tissues and organoid culture (Cao et al., 2012; Kuppusamy et al., 2015; Lin et al., 2017; Machiraju and Greenway 2019; Maddah et al., 2015; Parikh et al., 2017; Ruan et al., 2016; Rysa et al. 2018; Trenerry et al. 2011; Ulmer et al., 2018; Uosaki et al., 2015; Varzideh et al., 2019; Yang et al. 2014; Yoshida et al., 2018). These techniques have demonstrated the potential of generating CMs that show more aligned sarcomere organization, improved contractility, enhanced action potential upstroke velocity, and a switch from aerobic glycolysis to oxidative phosphorylation. Although the aforementioned strategies promote maturity of these cells structurally, physiologically, and metabolically, limitations such as scalability, cellular damages, and technical challenges remain to be resolved (Jiang et al., 2018).

To understand the underlying mechanisms governing CM maturation, we utilized a metabolic reporter to facilitate the purification of stem cell-derived CMs at various physiological states (fetal or matured). It is known that, during early cardiac development, glycolysis is the major source of energy for fetal CMs. As CMs mature, mitochondrial oxidative phosphorylation becomes the major source of energy for the heart. Leveraging on the role of hexokinase-1 (HK1), the first enzyme to phosphorylate glucose in the glycolysis pathway, we generated a metabolic reporter cell line (H7 HK1-GFP) to track on the metabolic shifts from glycolysis to β-oxidation, which occurs

 © 2021 The Authors.
This is an open access article under the CC BY license (http://creativecommons.org/licenses/by/4.0/).

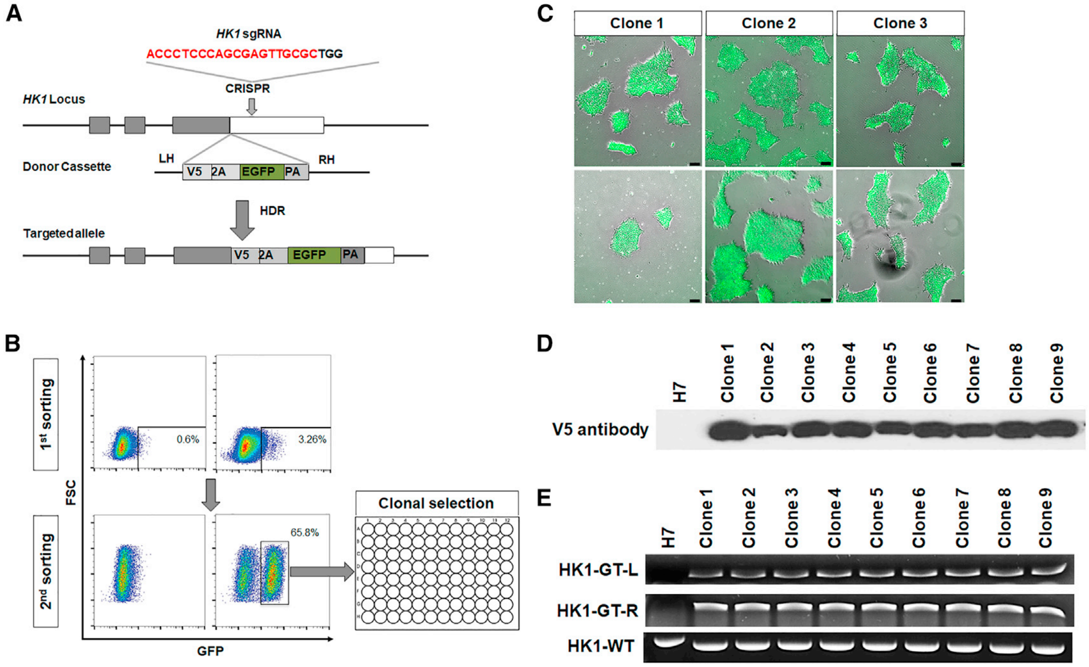

Figure 1. Generation of CRISPR-Cas9-mediated knockin H7 HK1-EGFP reporter

(A) Schematic illustration of the generation of the knockin reporter into the HK1 locus. HK1 sgRNA mediated a double-strand break within the HK1 locus replacing the stop codon with a donor cassette V5-2A-EGFP sequence in frame.

(B) Clonal expansion of H7 HK1-GFP knockin reporter cell. hESCs (H7) were electroporated with HK1 sgRNA and donor vector. The first round of fluorescent-activated cell sorting for GFP-positive cells showed 3.26% efficiency. The second round of fluorescent-activated cell sorting performed to enhance the clonal efficiency showed 65.8% efficiency.

(C) Representative images of H7 HK1-GFP clones. Green fluorescent colonies reflect the expression of HK1 gene in these clones. Scale bar, 100 μm.

(D) Western blot showing nine H7 HK1-GFP clones harboring the V5-2A-EGFP knockin sequence, which was absent in wild-type H7 ESCs. Cell lysates were probed with antibody against the V5 tag.

(E) PCR genotyping results indicated that all nine clones possess the V5-2A-EGFP knockin sequence.

during CM maturation (Jiang et al., 2018). Thus, this reporter cell line could facilitate the isolation of fetal-like CMs and relatively more matured CMs, based on their GFP expression level. By performing RNA sequencing on both fetal and matured CMs, we identified the upregulation of the interferon-signaling pathway, which is mediated by the JAK-STAT pathway, to be important for CM maturation. The findings were further validated by IFN-γ treatment in the purified population of fetal-like CMs, where increased pSTAT1 expression was observed. This in turn resulted in an enhancement in structural maturation of treated hPSC-CMs as both sarcomere length and myofibril width increased significantly. Functionally, IFN-γ-treated hPSC-CMs also exhibited electrophysiological properties corresponding to relatively more matured CMs compared with the untreated population. Collectively, this study highlights the feasibility of scaling up the acceleration of the maturation process of hPSC-CMs by IFN-γ treatment.

RESULTS

Generation of CRISPR-Cas9-mediated knockin H7 HK1-GFP reporter cells

The HK1-GFP reporter line was generated using the H7 human embryonic stem cell (hESC) line. In brief, the *HK1* stop codon was replaced by a V5-2A-EGFP sequence, resulting in an in-frame V5 tag and bicistronic 2A-EGFP via CRISPR-Cas9 HK1-sgRNA-guided homologous repair (Figure 1A). Fluorescence-activated cell sorting (FACS) was performed twice to purify ESCs harboring the gene insert based on their GFP expression levels. This was followed by single-cell isolation and expansion to achieve clonal HK1-GFP-positive clones (Figure 1B). We successfully expanded nine GFP-positive hESC clones; three of which (C1, C2, and C3) were selected for this study (Figure 1C). The three H7-HK1-GFP clones were shown to be karyotypically normal (Figure S1). We further performed western blot to confirm that the knockin of V5-2A-EGFP

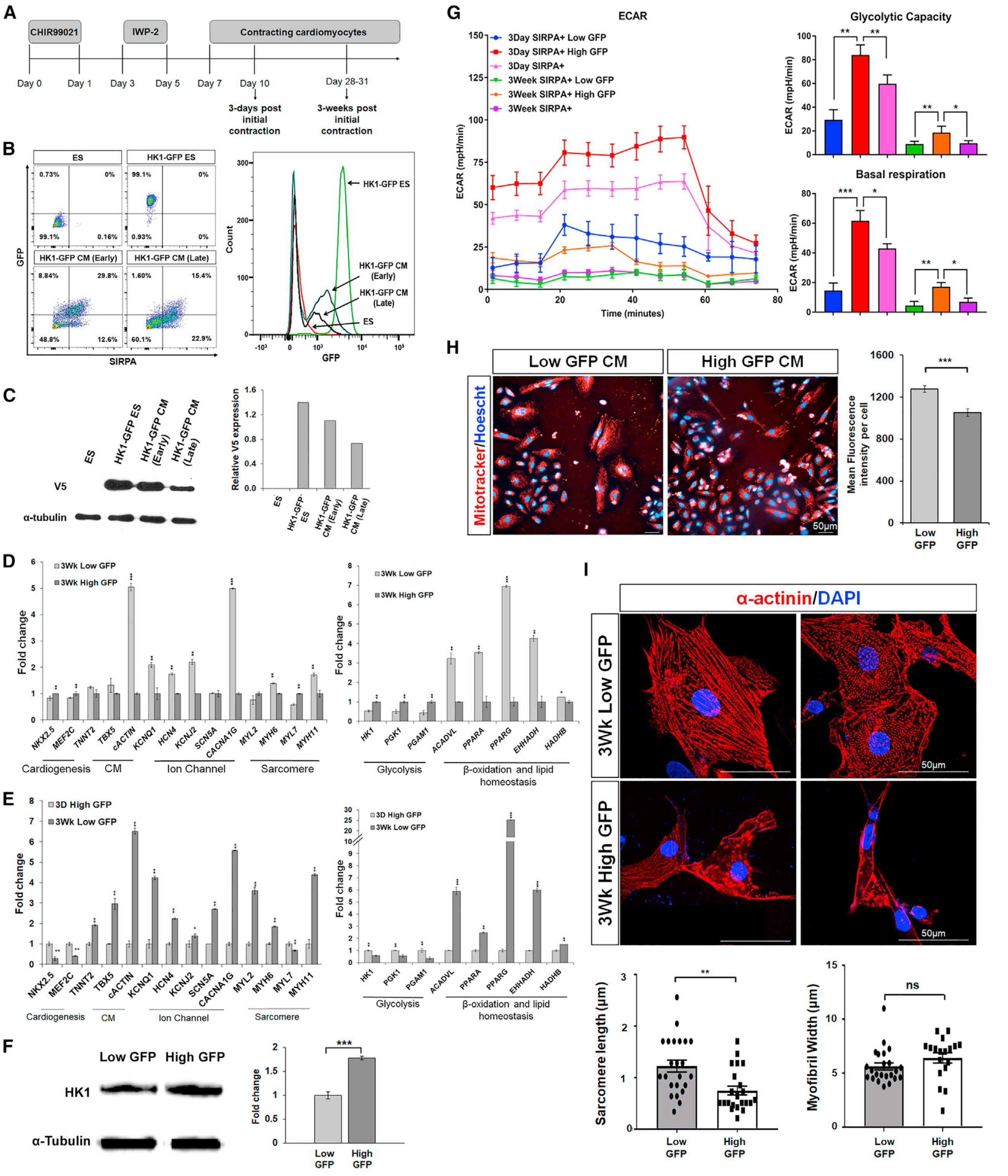

Figure 2. Reporter cell line reflects metabolic shift observed in maturing CMs

(A) Schematic diagram illustrating the differentiation protocol adopted to generate CMs from human embryonic stem cells. The differentiated CMs were analyzed at 3 days and 3 weeks post-initial contraction.

(B) Representative flow cytometry analysis of SIRPA+ CMs derived from H7 HK1-GFP reporter. Wild-type ESCs and HK1-GFP ESCs were used as a negative control. Percentages of SIRPA+/low- and high-GFP-expressing cells were compared between wild-type ESCs, HK1-GFP

(legend continued on next page)

sequence was successful in all nine ESC clones (Figure 1D). Polymerase chain reaction (PCR) genotyping also validated that the clones have the correct knockin reporter sequences in their genomes (Figure 1E). These results demonstrated the accuracy and purity of the knockin sequence V5-2A-GFP in our H7 HK1-GFP metabolic reporter cells.

Reporter cell line reflects metabolic shift observed in maturing CMs

To validate the utility of the reporter cell line, H7 HK1-GFP ESCs were differentiated into CMs using an established differentiation protocol and the differentiated CMs were analyzed at 3 days and 3 weeks post-initial contraction (Figure 2A) (Lian et al., 2013). Firstly, we demonstrated that wild-type hESCs do not express GFP (Figure 2B). As expected, flow cytometric analysis revealed that most, if not all, of the HK1-GFP hESCs expressed high levels of GFP (Figure 2B). Notably, the number of high-GFP-expressing cells decrease as hESCs differentiate to early fetal CMs (SIRPA+). A further reduction from 29.8% to 15.4% of high-GFP-expressing CMs was observed as the CMs were allowed to mature for another 3 weeks (late CMs) (Figure 2B). We further validated the shift in glycolysis by performing western blot with anti-V5-tag antibody, which showed decreasing V5 expression as hESCs differentiate to early CMs (3 days) and later mature into adult CMs (3 weeks) (Figure 2C).

As CM differentiation is an asynchronous event, we suspected 3-week low- and high-GFP-expressing CMs represent two distinct populations of cells with varying maturation status. Hence, we investigated whether purified sub-populations of CMs accurately reflects the physiological maturation status (fetal, high GFP; matured, low GFP) by examining genes that are important during cardiac maturation, such as ion channels, sarcomere, and metabolism genes (Uosaki et al., 2015; Yang et al. 2014). Flow cytometric sorting was performed on 3-day and 3-week post-initial contracting CMs as illustrated in Figure S2. As expected, quantitative gene expression revealed that the FACS-sorted 3-week low-GFP sub-group displayed elevated expression of ion channel (*KCNQ1*, *HCN4*, *KCNJ2*, *SCN5A*, and *CACNA1G*) and sarcomere genes (*MYL2*, *MYH6*, *MYL7*, and *MYH11*), while down-regulation of early cardiogenesis genes (*NKX2.5* and *MEF2C*) were observed compared with the 3-week high-GFP CMs (Figure 2D). Consistently, 3-week low-GFP CMs displayed higher levels of β-oxidation genes (*ACADVL*, *PPARA*, *PPARG*, *EHHADH*, and *HADHB*) and lower expression of glycolytic genes (*HK1*, *PGK1*, and *PGAM1*) in comparison with 3-week high-GFP CMs (Figure 2D). These aforementioned differences in gene expressions during CM maturation were more pronounced between the 3-week low-GFP CMs and 3-day high-GFP CMs (Figure 2E). Accordingly, higher HK1 protein expression (~1.7-fold) was observed in 3-week high-GFP CMs compared with the 3-week low-GFP

ESCs, 3-day post-initial contracting HK1-GFP CMs (early) and 3-week post-initial contracting HK1-GFP CMs (late). Representative histogram showing shifts in GFP expression as H7 HK1-GFP-derived cells differentiate to CMs. The experiments were repeated three times.

(C) Immunoblot showing decreasing V5 expression as H7 HK1-GFP reporter cells differentiate into early (3 days) and late (3 weeks) CMs. Graphical quantification of HK1-V5 protein expression as H7 HK1-GFP reporter cells differentiate into fetal and matured CMs. The experiments were repeated three times.

(D) Quantitative PCR analysis illustrated increased mRNA transcript expression of CMs, ion channel, sarcomere, β-oxidation, and lipid homeostasis genes, with decreased expression of cardiogenesis and glycolysis genes in 3-week post-initial contraction (expressing low GFP) compared with 3-week post-initial contraction (expressing high GFP) CMs. Data are represented as fold-change normalized to control samples, while expression of each gene was normalized to β-actin.

(E) Quantitative PCR analysis illustrated increased mRNA transcript expression of CMs, ion channel, sarcomere, β-oxidation, and lipid homeostasis genes, with decreased expression of cardiogenesis and glycolysis genes in 3-week post-initial contraction (expressing low GFP) compared with 3-day post-initial contraction (expressing high GFP) CMs. Data are represented as fold-change normalized to control samples, while expression of each gene was normalized to β-actin. For (D) and (E), data are presented as mean ± SD, n = 3 independently differentiated groups. Data information: statistical analysis was performed using Student's two-tailed t test. *p < 0.05, **p < 0.01, ***p < 0.001.

(F) Western blot and densitometric analysis shows higher HK1 protein expression in 3-week high-GFP-expressing CM compared to 3-week low-GFP-expressing CM. Data are presented as mean ± SD, n = 3 independently differentiated groups. Data information: Statistical analysis was performed using students two-tailed t test. ***p < 0.001.

(G) ECAR measurements using glycolysis stress assay was performed on 3-day and 3-week post-initial contracting CMs. Measurement of basal glycolysis and glycolytic capacity of (1) those expressing SIRPA+/low GFP, (2) SIRPA+/high GFP, and (3) SIRPA+ population only. Data represent mean ± SD, n = 3 independently differentiated groups.

(H) Immunostaining of low-/high-GFP-expressing CMs with MitoTracker (red) that stains mitochondria in live cells with Hoescht (blue). Mitochondrial content was measured based on mean fluorescence intensity per cell. Data represent mean ± SEM, n = 3 independently differentiated groups. Scale bars, 50 μm.

(I) Immunostaining and graphical quantification of sarcomere length (μm) (n = 40, p = 0.0002) and myofibril width (μm) (n = 40, p = 0.1664) in low-/high-GFP-expressing CMs stained with α-actinin (red). Nuclei were stained in blue with DAPI. Data represent mean ± SD, n = 25 cells from three independently differentiated groups. Scale bars, 50 μm. Statistical analysis was performed using Student's two-tailed t test. *p < 0.05, **p < 0.01, ***p < 0.001.

CMs, verifying that GFP expression is, indeed, indicative of HK1 expression (Figure 2F). These results validate the potential of the H7 HK1-GFP reporter cell line as a useful tool to track the physiological status of CMs.

To further illustrate that CMs derived from the H7 HK1-GFP reporter line can be separated into fetal or matured CMs based on their GFP expressions, we performed a glycolytic stress test on three sub-populations of FACS-sorted CMs: (1) those expressing SIRPA+/low GFP, (2) SIRPA+/high GFP, and (3) SIRPA+ population only. Glycolysis, measured by extracellular acidification rate (ECAR), revealed that SIRPA+/high-GFP-expressing CMs, 3-day post-initial contraction, exhibited the greatest ECAR for basal respiration and glycolytic capacity compared with SIRPA+/low GFP and SIRPA+ population only (Figure 2G). Expectedly, the ECAR profile of CMs from 3-week post-initial contraction is much lower than CMs that are 3 days post-initial contraction, indicating that the 3-week post-initial contracting CMs are metabolically more matured than the 3-day post-initial contracting CMs. Importantly, within the same time point (either 3-day or 3-week post-initial contracting CMs), the metabolic reporter was capable of enabling isolation of a specific population of CMs that represents it metabolic status.

Expectedly, due to the higher bioenergetic demands of matured CMs, live staining of mitochondria in CMs using MitoTracker also revealed significantly higher (20%) mitochondrial content in low-GFP-expressing CMs compared with high-GFP-expressing CMs, as demonstrated by mean fluorescence intensity ($p < 0.001$) (Figure 2H). In addition, low-GFP-expressing CMs were also found to have longer sarcomere length ($\geq$50%) ($p < 0.001$), although a wider myofibril width was not observed (Figure 2I). Henceforth, the results demonstrated that low HK1-GFP CMs possess relatively more matured bioenergetics and sarcomeric structure compared with high HK1-GFP CMs at the same time point.

Upregulation of interferon-signaling pathways promotes CM maturation

To identify molecular mechanisms governing the glycolytic shift that is associated with CM maturation, RNA sequencing was performed on 3-day (fetal-like CMs expressing high GFP) and 3-week (relatively more matured CMs expressing low GFP) post-initial contracting CMs. Gene set enrichment analysis showed downregulation of genes involved in glycolysis and gluconeogenesis in the 3-week post-initial contracting CMs (low GFP expressing) compared with 3-day post-initial contracting CMs (high GFP expressing) (Figure S3). Gene ontology and KEGG pathway enrichment analysis revealed upregulation of gene clusters related to interferon signaling and neutrophil degranulation in the matured CMs ($p < 0.05$, FDR < 0.1) (Figure 3A). Conversely, genes associated with cell-cycle checkpoints and DNA replication were downregulated ($p < 0.05$, FDR < 0.1) (Figure 3B). The top 24 up- and down-regulated transcription factors (TFs) in the aforementioned pathways are as shown in Figure 3C. A comprehensive list of differentially expressed genes analyzed is provided in Tables S3, S4, and S5. Next, visualization of functional enrichment was achieved via Cnetplot. The results validated the interaction of genes involved in the interferon-signaling pathway, as shown by upregulated expression of TFs, such as *STAT1*, *IRF1*, *IRF2*, *IRF5*, and *IRF6* (Figure 3D).

Given the implications of the JAK-STAT pathway in promoting proliferation and preventing premature differentiation of myoblasts (Trenerry et al. 2011), this prompted us to investigate the potential role of *STAT1* during CM maturation. We utilized IFN-γ, an important regulator of immunity and inflammation to induce activation of the JAK-STAT pathway in PSC-derived CMs. A schematic of the treatment process is summarized in Figure 4A. Purified fetal CMs were isolated and treated with 25 ng/mL of IFN-γ. The results demonstrated upregulated mRNA expression of *STAT1* and its downstream targets, such as interferon response factors (*IRFs*) and interferon-stimulated genes in H7- and ES03-derived CMs (Figure 4B). Consistently, a significant increase in pSTAT1 and STAT1 protein was observed after 1, 4, and 8 h of IFN-γ treatment (Figures 4C and 4D). To assess the prolonged effects of IFN-γ on CM maturation, we investigated the relative mRNA expression levels of IFN-γ-treated CMs after 3, 7, and 10 days. Increased mRNA expression of sarcomere genes (*TNNT2*, *TNNT3*, *cACTIN*, *MYH6*, *MYL7*, and *MYH7*) was observed by day 7 in H7- and ES03-derived CMs (Figures 4E and S4A). These aforementioned differences in gene expression between IFN-γ-treated CMs and control were more pronounced after 10 days of IFN-γ treatment. Quantitative PCR also revealed increased mRNA expression of ion channel genes, mainly potassium and sodium channels, such as *KCNJ2*, *SCN3B*, and *SCN5A* (Figures 4F and S4B). This corresponded with increased expression of β-oxidation genes (*ACADVL* and *EHHADH*) (Figures 4G and S4C). Consistently, IFN-γ-treated H7-HK1-derived CMs demonstrated increased ratio of low:high GFP expression after 3 days (1.13-fold), 7 days (1.67-fold), and 10 days (2.76-fold), illustrating the lowering dependence on glycolysis as the cells mature (Figures 4L and S5A). Western blot showed increased expression of pSTAT1 and STAT1 in IFN-γ-treated groups, this was accompanied by increased relative expression of the MYH7:MYH6 ratio in 10-day IFN-γ-treated H7- and ES03-derived CMs compared with control (Figures 4H and 4I). Increased expression of MYH7 was further validated by flow cytometric analysis, which revealed an increased percentage of MYH7-positive CMs from 51.6% (control) to 60.6% (IFN-γ treated), and 28.93% (control)

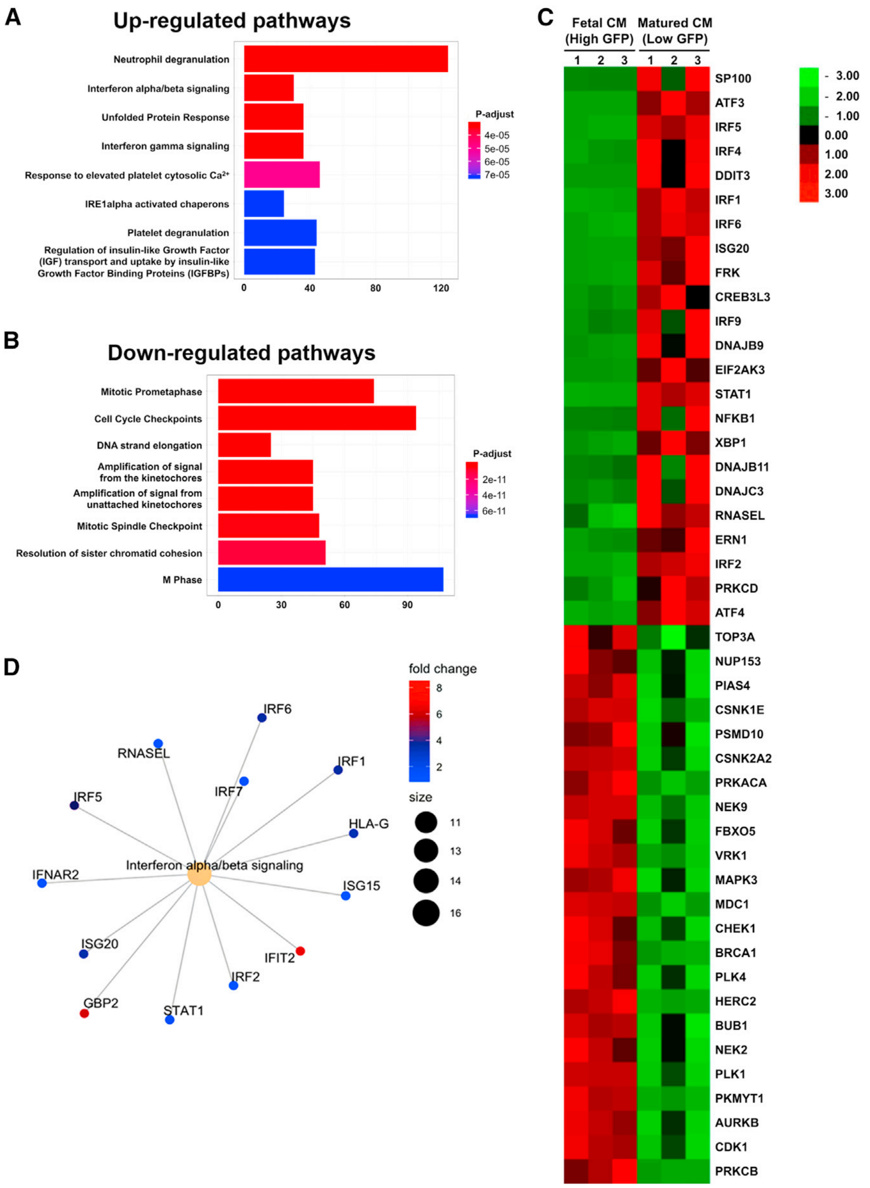

Figure 3. Bulk RNA sequencing analysis of differential expressed genes and pathways during CM maturation

(A and B) Gene ontology pathway enrichment analysis identified (A) upregulation and (B) downregulation of key pathways associated with matured CMs (low GFP) compared with fetal CMs (high GFP).

(C) Correlated clustered heatmap of a list of top 24 transcription factors in the top 8 up-/downregulated pathways and matured (low GFP) CMs compared with fetal CMs (high GFP). The color intensity represents column *Z* score, with red indicating high expression and green indicating low expression. For (A)–(C), n = 3 independently differentiated groups, $p < 0.05$, FDR < 0.1.

(D) CNet plot identified the interaction of genes involved in the interferon-signaling pathway. The color intensity represents fold-change.

to 32.57% (IFN-γ treated) in H7-derived CMs and ES0S3-derived CMs, respectively (Figures 4J and 4K).

To functionally characterize the effects of IFN-γ treatment on the CMs, 10-day post IFN-γ-treated CMs were stained with α-actinin, and parameters such as sarcomere length and myofibril width were quantified using ImageJ (Spletter et al., 2018). The results showed significant increase in both sarcomere length (2.209–2.388 μm, p = 0.0377) and myofibril width (4.788–5.287 μm, p = 0.042) in IFN-γ-treated H7-derived CMs (Figure 5A), as well as increased sarcomere length (1.285–1.333 μm, p = 0.0451) and increased sarcomere width (4.877–5.048 μm, p = 0.0153) in ES03-derived CMs (Figure 5B). In addition, the sarcomeric architecture of CMs was assessed using a computational tool, ZlineDetection, as described by Morris et al. (2020). CMs treated with IFN-γ for 10 days were stained with phalloidin and α-actinin, and confocal microscopic images were computationally assessed to compute the fraction of α-actinin composed of well-formed Z-lines and the skewness of the continuous Z-line (CZL). H7-derived CMs treated with IFN-γ demonstrated increased Z-line fraction (0.3749–0.4292, p = 0.0391) with no significant difference in skewness of CZL (Figure 5C). Similarly, we observed a significant increase in Z-line fraction (0.4652–0.5003, p = 0.0413) in IFN-γ-treated ES03-derived CMs compared with control. This was accompanied with

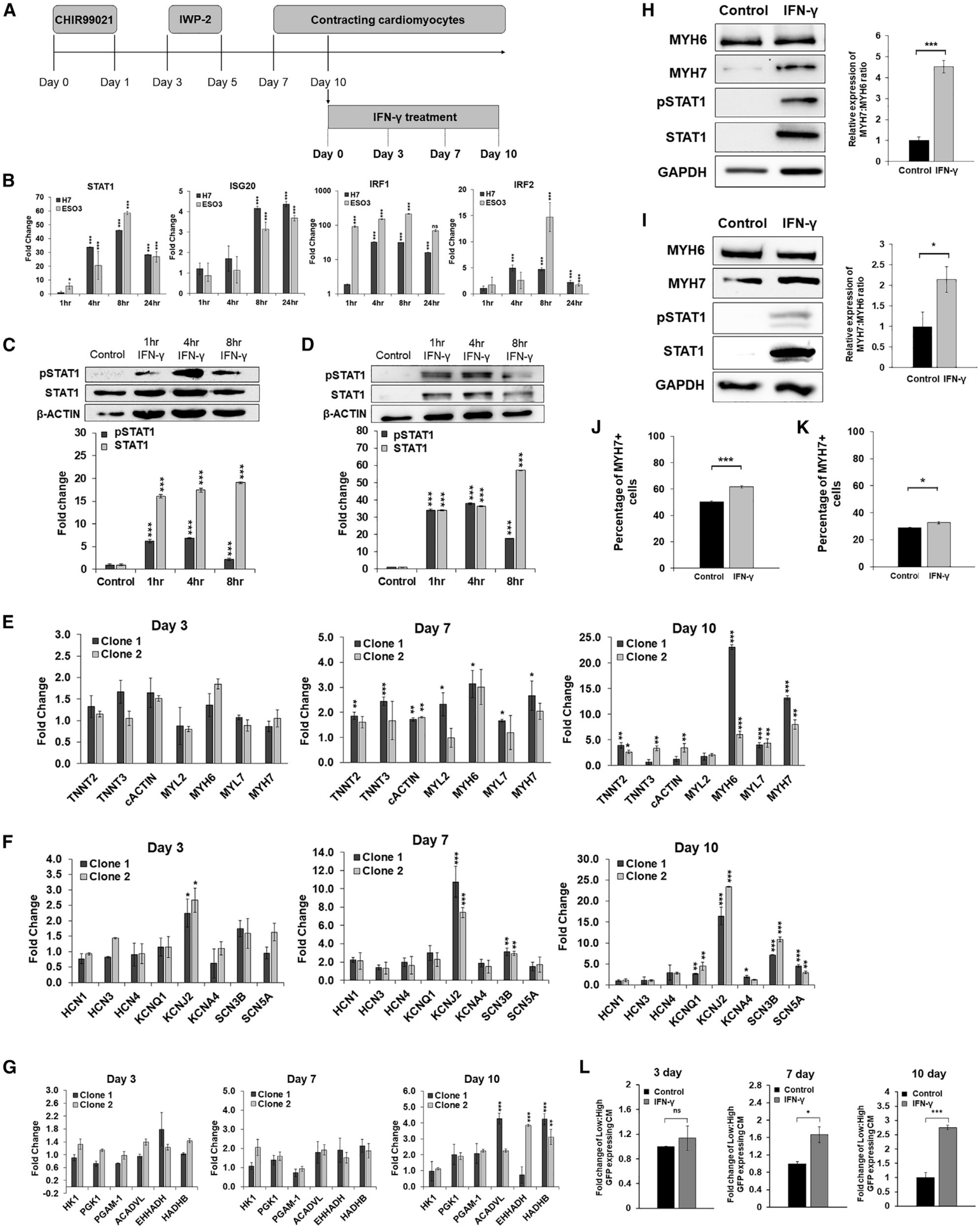

(legend on next page)

decreased skewness of CZL (2.09–1.915, $p = 0.0406$) (Figure 5D), hence suggesting relatively more aligned sarcomeres associated with a relatively more matured state.

IFN-γ-treated hESC-derived CMs exhibit functionally more matured electrophysiology

Functional assessment of CM electrophysiology was performed by knocking in GCaMP6s, a genetically encoded calcium indicator protein (GCaMP6s), in both H7- and ES03-derived CMs (Maddah et al., 2015; Kaestner et al., 2014; Huebsch et al., 2015). Fetal CMs that were isolated on day 7 of differentiation were treated with IFN-γ for 3, 7, and 10 days (Figure 4A). As illustrated in Figure 6A, the GCaMP6s reporter cell line provides high-throughput analysis of the mean calcium transient duration, calcium transient peak intensity, depolarization speed (also known as upstroke velocity), and repolarization duration of each contraction. Consistent with other studies, our data showed that IFN-γ-treated H7- and ES03-derived CMs demonstrated increased calcium peak intensity and calcium transient duration (Figures 6B, 6C, S6A, and S6B). This is in accordance with earlier work by Yang et al. (2019) that demonstrated enhanced calcium transient peak height (intensity), and increased action potential upstroke velocity, during maturation of hPSC-derived CMs (Guo and Pu 2020; Yang et al., 2019). As increased upstroke velocity is a critical distinguishing property of matured CMs (Jiang et al., 2018), we investigated the effect of IFN-γ treatment on the depolarization speed of CMs. The results demonstrated a significant increase in depolarization speed, which was coupled with delayed repolarization duration in IFN-γ-treated H7- and ES03-derived CMs compared with control (Figures 6D, 6E, S6C, and S6D). Representative videos showed that calcium transients demonstrated a faster depolarization speed in IFN-γ-treated H7-derived CMs, as shown by a steeper upstroke velocity (Videos S1 and S2).

Mechanistically, we demonstrated that IFN-γ exerts its effect through the JAK/STAT pathway by treating CMs with a JAK-STAT inhibitor, ruxolitinib. As shown in Figures 6B–6E and S6A–S6D, IFN-γ + 0.1 μM ruxolitinib treatment did not significantly attenuate the effects of IFN-γ in improving calcium transient duration, increasing peak calcium transient intensity, as well as increased depolarization speed and delayed repolarization duration. However, a significant reduction in these effects was observed in the IFN-γ + 1 μM ruxolitinib-treated group (Figures 6B–6E and S6A–S6D), suggesting that the inhibitor was capable of attenuating the effects of IFN-γ in a dose-dependent manner, as demonstrated in both H7- and ES03-derived CMs (Figures 6B–6E and S6A–S6D).

To further support our findings that IFN-γ promotes functional maturation of hESC-derived CMs, we employed a multielectrode array (MEA) system to investigate the cellular electrophysiology of IFN-γ-treated CMs. The results from Figures 7 and S7 demonstrated that IFN-γ-treated CMs exhibited a relatively more matured phenotype compared with control CMs. This was shown by increased peak-to-peak amplitude, increased upstroke velocity, and longer RR interval (beat period) in H7-, ES03-, and G608G

Figure 4. Upregulation of the JAK-STAT pathway is important for CM maturation

(A) Schematic diagram illustrating the differentiation protocol utilized to generate CMs from hESCs. The contracting CMs were used to investigate the effects of IFN-γ treatment at various time points (3, 7, and 10 days).

(B) Quantitative PCR analysis illustrated increased mRNA transcript expression of *STAT1*, *IRF1*, *IRF2*, and *ISG20* in 1–24 h IFN-γ-treated H7- and ES03-derived CMs compared with the control of the respective cell line. Data are represented as fold-change normalized to β-actin. Data are presented as mean ± SD, n = 3 independently differentiated groups. Data information: statistical analysis was performed using Student's two-tailed t test. $^{*}p < 0.05$, $^{**}p < 0.01$, $^{***}p < 0.001$, $^{***}p < 0.0001$.

(C and D) Western blot shows 1–8 h IFN-γ-treated hESC-derived CMs (left, H7 cell line; right, ES03 cell line). Cell lysates were probed with pSTAT1 and STAT1. Densitometric analysis showing relative protein expression of pSTAT1 and STAT1 normalized to control samples, after normalization to β-actin. Data are presented as mean ± SD, n = 3 independently differentiated groups.

(E–G) Quantitative PCR analysis illustrated increased mRNA transcript expression of (E) sarcomere, (F) ion channel, and (G) metabolic genes, in IFN-γ-treated H7-derived CMs (clones 1 and 2) compared with control. Data are represented as fold-change normalized to control samples, while expression of each gene was normalized to β-actin. Data are presented as mean ± SD, n = 3 independently differentiated groups. Data information: statistical analysis was performed using Student's two-tailed t test. $^{*}p < 0.05$, $^{**}p < 0.01$, $^{***}p < 0.001$.

(H and I) Western blot shows 10-day post IFN-γ-treated (H) H7-derived CMs and (I) ES03-derived CMs compared with control. Cell lysates were probed with STAT1, pSTAT1, MYH7, MYH6, and GAPDH. Graphical representation of the ratio of MYH6:MYH7 presented as fold-change normalized to GAPDH. Data are presented as mean ± SD, n = 3 independently differentiated groups. Data information: statistical analysis was performed using Student's two-tailed t test. $^{***}p < 0.0001$.

(J and K) Flow cytometry analysis shows increased percentage of MYH7-positive cells, 10-day post IFN-γ treatment in (J) H7- and (K) ES03-derived CMs compared with control. Data are presented as mean ± SEM, n = 3 independently differentiated groups. Data information: statistical analysis was performed using Student's two-tailed t test. $^{*}p < 0.05$, $^{**}p < 0.01$, $^{***}p < 0.001$.

(L) Bar graph showing increased ratio of low-/high-GFP-expressing CMs in 10-day post IFN-γ-treated H7-derived CMs compared with control. $^{*}p < 0.05$, $^{**}p < 0.01$, $^{***}p < 0.001$. Data are presented as mean ± SEM, n = 3 independently differentiated groups.

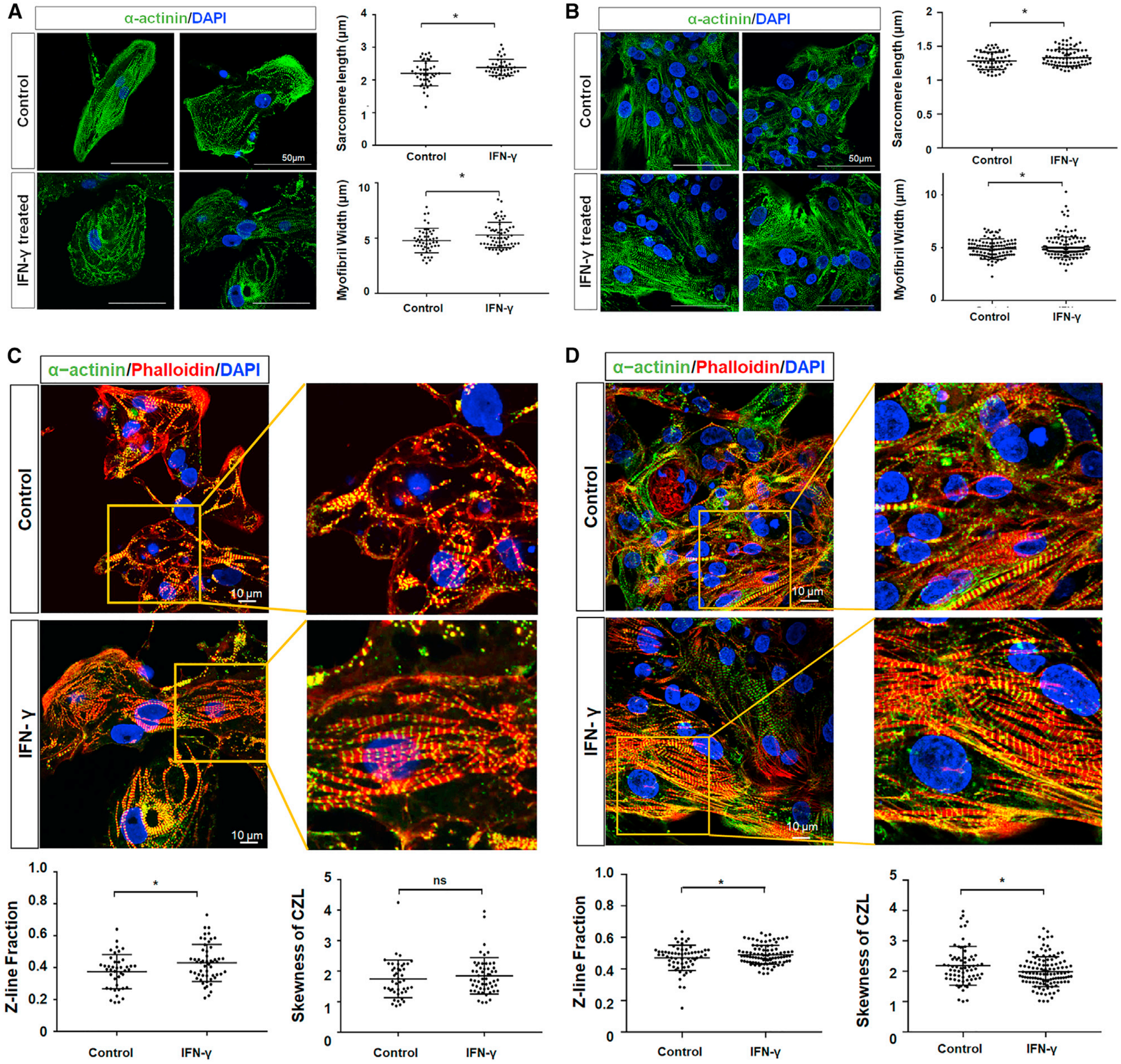

Figure 5. Improved sarcomeric structure in IFN-γ-treated H7- and ES03-derived CMs

(A and B) Representative image and graphical quantification of sarcomere length (μm) and myofibril width (μm) of control and 10-day post IFN-γ-treated (A) H7- and (B) ES03-derived CMs compared with control group. CMs were stained with α-actinin (green). Nuclei were stained in blue with DAPI. H7- and ES03-derived CMs, data represent mean ± SD, n = 50–80 cells from 30 microscopic fields, 3 independently differentiated groups. Scale bars, 50 μm.

(C and D) Representative image and graphical quantification of Z-line fraction and skewness of CZL in 10-day post IFN-γ-treated (C) H7- and (D) ES03-derived CMs compared with control group. CMs were stained with α-actinin (green) or phalloidin (red). Nuclei were stained in blue with DAPI. H7- and ES03-derived CMs, data represent mean ± SD, n = 50–80 cells from 30 microscopic fields, 3 independently differentiated groups. Scale bars, 10 μm. *$p < 0.05$.

hiPSC-derived fetal CMs (Figures 7 and S7). These finding are in accordance with earlier studies reporting increased conductance velocity and beat period in prolonged culture of hiPSC-CMs (Kumar et al., 2019). In addition, as CMs mature, their action potentials change dramatically, including loss of automaticity, acquiring a more negative resting membrane potential (approximately −90 mV) (Karbassi et al., 2020). Hence, the greater peak-to-peak amplitude

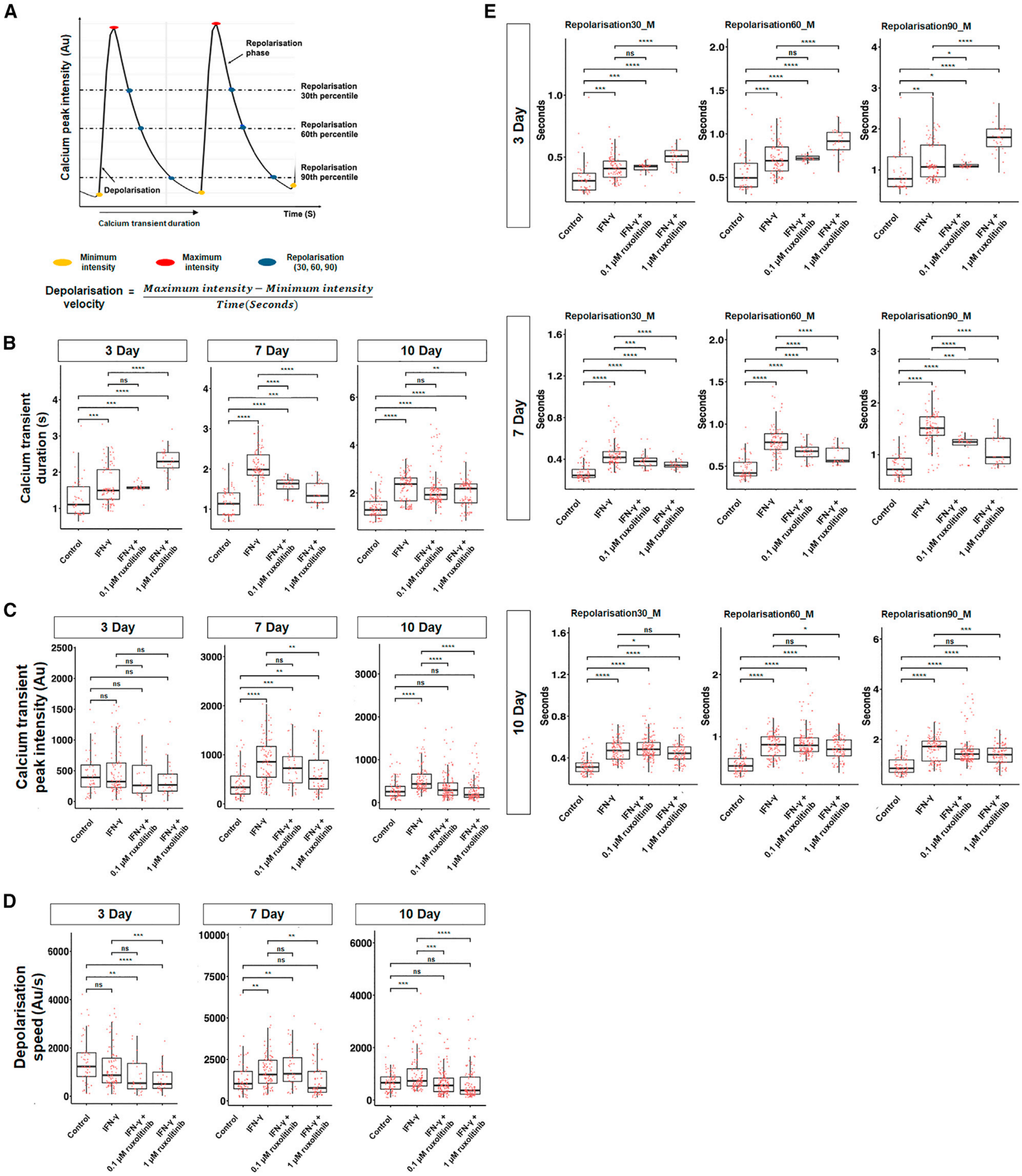

Figure 6. Electrophysiological assessment of calcium transients in IFN-γ and ruxolitinib-treated H7-GCaMP6s-derived CMs

(A) Representative electrophysiological image of H7-GCaMP6s expressing CMs recorded 3, 7, and 10 days post IFN-γ treatment.

(B)–(E) Representative data of one of the two H7-GCaMP6s clone-derived CMs that were differentiated and analyzed. Dot plot showing mean (B) calcium transient duration (s), (C) calcium transient peak intensity (Au), (D) depolarization speed (Au/s), and (E) repolarization duration (30th, 60th, and 90th percentiles) per cell, measured across 30 s, n = 50 cells, three independently differentiated groups, two clones per cell line (H7). Results obtained using the ES03 cell line are presented in Figure S6. Data information: statistical analysis was performed using Student's two-tailed t test. *p < 0.05, **p < 0.01, ***p < 0.001, ****p < 0.0001.

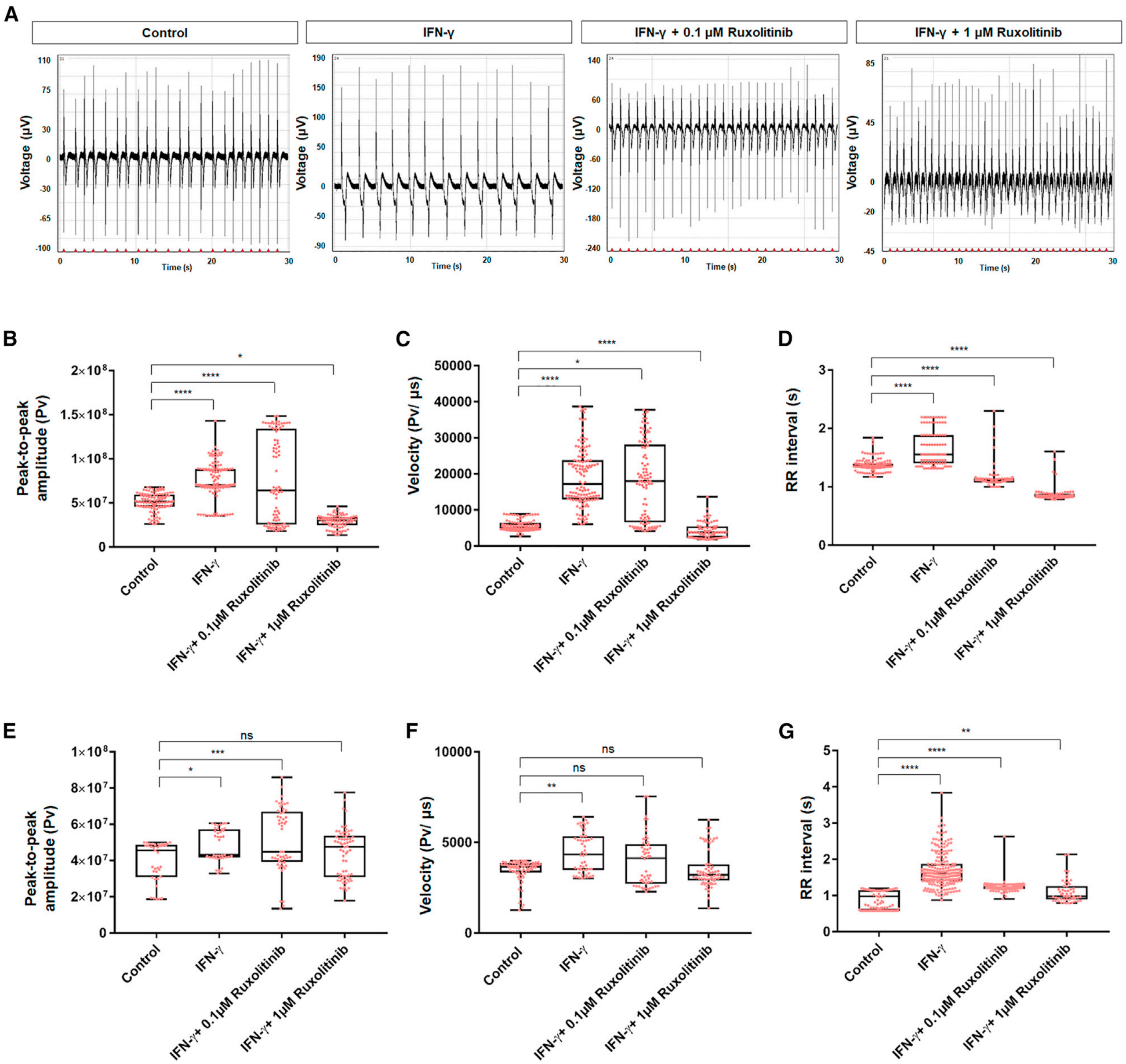

Figure 7. Electrophysiological assessment of IFN-γ-treated fetal CMs using a high-throughput multielectrode array system

(A) Representative extracellular field potential recordings of H7-derived CMs; control, IFN-γ-treated, IFN-γ + 0.1 μM ruxolitinib, and IFN-γ + 1 μM ruxolitinib-treated groups, recorded 10 days after treatment.

(B–D) Dot plot showing mean peak-to-peak (B) amplitude (pV), (C) velocity (pV/ms), and (D) RR interval of H7-derived CMs (10 days post IFN-γ treatment) measured across 5 min, n = 3 independently differentiated groups, two clones were used. *$p < 0.05$, **$p < 0.01$, ***$p < 0.001$, ****$p < 0.0001$.

(E–G) Dot plot showing mean peak-to-peak (E) amplitude (pV), (F) velocity (pV/ms), and (G) RR interval of ES03-derived CMs (10 days post IFN-γ treatment) measured across 5 min, n = 3 independently differentiated groups, two clones were used. *$p < 0.05$, **$p < 0.01$, ***$p < 0.001$, ****$p < 0.0001$.

observed in the IFN-γ-treated group suggests a more drastic change in resting membrane potential (measuring amplitude between the maximum and the minimum of a Na peak). Consistently, the aforementioned inhibitory effect exerted by ruxolitinib treatment was observed in MEA (Figures 7B–7G and S7A–S7C). In the presence of ruxolitinib (0.1 and 1 μM), the effects of IFN-γ treatment was nullified in a dose-dependent manner in both H7-, ES03-, and G608G

hiPS-derived fetal CMs (Figures 7B–7G and S7A–S7C). Hence, this demonstrated the role of the JAK-STAT pathway in regulating CM maturation and electrophysiological function through IFN-γ activation.

DISCUSSION

While hPSC-CMs hold great promise in cardiac disease modeling and cell-based therapies, the CMs differentiated *in vitro* often exhibit fetal-like characteristics. The immaturity of hPSC-CMs would inevitably result in poor cell-based therapy outcomes, inaccurate disease modeling and drug screening, thus leading to unsuccessful clinical translations.

Herein, we demonstrated the capability of a HK1-GFP metabolic reporter to isolate CMs at different physiological states. While glycolysis and gluconeogenesis genes were found to be downregulated in 3-week post-initial contracting CMs compared with 3-day post-initial contracting CMs (Figure S2), they were, however, not ranked as the top differentially expressed pathways in the RNA sequencing data. Hence, metabolic pathways were not included in Figures 3A and 3B. This study presents a relatively straightforward method to enhance both functional and structural maturation of hESC-derived CMs by modulating JAK-STAT signaling through the use of IFN-γ. While neutrophil degranulation was presented as the top differentially expressed pathway, we chose to investigate the interferon-signaling pathway and its potential in promoting CM maturation as upregulation of STAT1 via the JAK-STAT pathway has been reported in cellular maturation in other systems (Heim 1999; Putz et al., 2016). For instance, earlier studies showed that the JAK-STAT pathway plays a key role in promoting myoblast proliferation and preventing premature differentiation via upregulation of genes associated to muscle maturation (Sun et al., 2007; Trenerry et al. 2011). Furthermore, IFN-mediated activation of the JAK-STAT pathway has also been shown to be an important regulator of natural killer cell, monocyte, and dendritic cell maturation (Coccia et al., 1999; Jackson et al., 2004; Putz et al., 2016). Similarly, with inhibitor studies, we demonstrated the significance of the JAK-STAT pathway in enhancing maturation of CMs, as exhibited through modulation of CM electrophysiological function. Interestingly, improved calcium dynamics and electrophysiological function was observed in IFN-γ-treated H7- and ES03-derived CMs, and these effects were reversed by co-administration with a JAK/STAT inhibitor, ruxolitinib, in particular after 7 and 10 days. The inhibitory effects of ruxolitinib on H7- and ES03-derived CMs was not observed 3 days after treatment as it functions to modulate cytokine-stimulated intracellular signaling by inhibiting JAK1 and JAK2. Thus, our data suggest that the inhibitory effects by ruxolitinib on JAK-STAT signaling may require prolonged exposure to elicit an intracellular effect on the transcription of STAT1 as suggested by other studies (Ostojic et al. 2012).

In our study, hESC-derived CMs treated with IFN-γ demonstrated a shift from MYH6 to MYH7 expression (Figures 4H and 4I), increased percentage of MYH7-expressing CMs (Figures 4J and 4K), increased sarcomere length and width (Figures 5A and 5B), and increased Z-line fraction (Figures 5C and 5D). In addition, a shift from high to low HK1-GFP expression (Figure 4L and S4A) was observed, suggesting a shift toward a relatively more matured metabolic profile. Furthermore, functional studies leveraging on calcium imaging demonstrated increased calcium transient duration (Figures 6B and S6A), increased calcium peak intensity (Figures 6C and S6B), faster depolarization speed (Figures 6D and S6C), and delayed repolarization duration (Figures 6E and S6D) when fetal CMs were treated with IFN-γ. Consistent with calcium imaging data, functional maturation of PSC-derived CMs exhibited improved electrophysiological profile as shown with MEA (Figures 7 and S7). Taken together, upon treatment with IFN-γ, both calcium signaling and extracellular field potential revealed improved calcium dynamics and enhanced contractile velocity, which are characteristics of relatively more matured CMs, as reported in earlier studies (Karbassi et al., 2020; Kumar et al., 2019; Pioner et al., 2019; Yang et al., 2019).

In summary, we identified a novel role of JAK-STAT signaling in hPSC-CM maturation. We showed that IFN-γ treatment activates the JAK-STAT pathway and upregulates *STAT1* expression in hPSC-derived fetal CMs, thereby promoting their structural and functional maturation. This biochemical approach provides a robust and viable option, with the potential of upscaling for the generation of functionally matured hPSC-derived CMs required for downstream translational processes.

EXPERIMENTAL PROCEDURES

Cell culture and media

The human cell lines were cultured in feeder-free conditions on culture plates coated with Matrigel matrix (Corning, USA) diluted in DMEM/F12 (Gibco, USA). Cells were maintained in StemMACS iPS-Brew XF basal medium supplemented with iPS-Brew XF, 50× supplement (Miltenyi Biotec, Germany) and were maintained in 37°C under humidified atmosphere of 5% CO_2. Culture medium was changed daily. After 80%–90% confluency was reached, hESCs were passaged using 1 mg/mL collagenase IV (Gibco). When setting up the hPSCs for directed differentiation, the cells were passaged using StemPro-Accutase (Gibco) as single cells with 5 μM of Y27632 (Miltenyi Biotec).

Data and code availability

RNA sequencing data generated in this study are made available at the Gene Expression Omnibus (GEO). The accession number for

the raw and processed data reported in this paper is GEO: GSE142826.

SUPPLEMENTAL INFORMATION

Supplemental information can be found online at https://doi.org/10.1016/j.stemcr.2021.10.009.

AUTHOR CONTRIBUTIONS

B.S.S., S.Y.N., and B.X.H. conceptualized and designed the study. B.X.H. performed most of the experiments. H.Y. generated CRISPR knockin cell line. P.S. and C.L.S. generated the human G608G iPSC line. J.H.H., J.K.S.P., L.C.L., C.Y.Y.L., and W.K.C. performed some of the experiments and data analysis. O.A. and H.H.Y. performed gene expression profiling and pathway enrichment analyses. B.X.H. and B.S.S. wrote the manuscript. All authors approved and read the manuscript.

DECLARATION OF INTERESTS

The authors declare no competing interests.

ACKNOWLEDGMENTS

This work is supported by the Agency for Science, Technology and Research (Singapore) and by a grant from the National Medical Research Council to B.S.S. (NMRC/OFYIRG/0017/2016). B.X.H., J.K.S.P., and J.H.H. are supported by the National University of Singapore graduate scholarships.

Received: March 31, 2021
Revised: October 14, 2021
Accepted: October 15, 2021
Published: November 11, 2021

REFERENCES

Cao, N., Liu, Z., Chen, Z., Wang, J., Chen, T., Zhao, X., Ma, Y., Qin, L., Kang, J., Wei, B., et al. (2012). Ascorbic acid enhances the cardiac differentiation of induced pluripotent stem cells through promoting the proliferation of cardiac progenitor cells. Cell Res. *22*, 219–236.

Chen, H.S., Kim, C., and Mercola, M. (2009). Electrophysiological challenges of cell-based myocardial repair. Circulation *120*, 2496–2508.

Coccia, E.M., Del Russo, N., Stellacci, E., Testa, U., Marziali, G., and Battistini, A. (1999). STAT1 activation during monocyte to macrophage maturation: role of adhesion molecules. Int. Immunol. *11*, 1075–1083.

Funakoshi, S., Miki, K., Takaki, T., Okubo, C., Hatani, T., Chonabayashi, K., Nishikawa, M., Takei, I., Oishi, A., Narita, M., et al. (2016). Enhanced engraftment, proliferation, and therapeutic potential in heart using optimized human iPSC-derived cardiomyocytes. Sci. Rep. *6*, 19111.

Guo, Y., and Pu, W.T. (2020). Cardiomyocyte maturation: new phase in development. Circ. Res. *126*, 1086–1106.

Heim, M.H. (1999). The Jak-STAT pathway: cytokine signalling from the receptor to the nucleus. J. Recept. Signal. Transduct. Res. *19*, 75–120.

Huebsch, N., Loskill, P., Mandegar, M.A., Marks, N.C., Sheehan, A.S., Ma, Z., Mathur, A., Nguyen, T.N., Yoo, J.C., Judge, L.M., et al. (2015). Automated video-based analysis of contractility and calcium flux in human-induced pluripotent stem cell-derived cardiomyocytes cultured over different spatial scales. Tissue Eng. Part C Methods *21*, 467–479.

Jackson, S.H., Yu, C.R., Mahdi, R.M., Ebong, S., and Egwuagu, C.E. (2004). Dendritic cell maturation requires STAT1 and is under feedback regulation by suppressors of cytokine signaling. J. Immunol. *172*, 2307–2315.

Jiang, Y., Park, P., Hong, S.M., and Ban, K. (2018). Maturation of cardiomyocytes derived from human pluripotent stem cells: current strategies and limitations. Mol. Cells *41*, 613–621.

Kaestner, L., Scholz, A., Tian, Q., Ruppenthal, S., Tabellion, W., Wiesen, K., Katus, H.A., Muller, O.J., Kotlikoff, M.I., and Lipp, P. (2014). Genetically encoded Ca^{2+} indicators in cardiac myocytes. Circ. Res. *114*, 1623–1639.

Karbassi, E., Fenix, A., Marchiano, S., Muraoka, N., Nakamura, K., Yang, X., and Murry, C.E. (2020). Cardiomyocyte maturation: advances in knowledge and implications for regenerative medicine. Nat. Rev. Cardiol. *17*, 341–359.

Kumar, N., Dougherty, J.A., Manring, H.R., Elmadbouh, I., Mergaye, M., Czirok, A., Greta Isai, D., Belevych, A.E., Yu, L., Janssen, P.M.L., et al. (2019). Assessment of temporal functional changes and miRNA profiling of human iPSC-derived cardiomyocytes. Sci. Rep. *9*, 13188.

Kuppusamy, M., Watanabe, H., Kasugai, S., and Kuroda, S. (2015). Effects of abutment removal and reconnection on inflammatory cytokine production around dental implants. Implant Dent *24*, 730–734.

Li, J., Minami, I., Shiozaki, M., Yu, L., Yajima, S., Miyagawa, S., Shiba, Y., Morone, N., Fukushima, S., Yoshioka, M., et al. (2017). Human pluripotent stem cell-derived cardiac tissue-like constructs for repairing the infarcted myocardium. Stem Cell Reports *9*, 1546–1559.

Lian, X., Zhang, J., Azarin, S.M., Zhu, K., Hazeltine, L.B., Bao, X., Hsiao, C., Kamp, T.J., and Palecek, S.P. (2013). Directed cardiomyocyte differentiation from human pluripotent stem cells by modulating Wnt/beta-catenin signaling under fully defined conditions. Nat. Protoc. *8*, 162–175.

Lin, B., Lin, X., Stachel, M., Wang, E., Luo, Y., Lader, J., Sun, X., Delmar, M., and Bu, L. (2017). Culture in glucose-depleted medium supplemented with fatty acid and 3,3,5-triiodo-l-thyronine facilitates purification and maturation of human pluripotent stem cell-derived cardiomyocytes. Front. Endocrinol. (Lausanne) *8*, 253.

Machiraju, P., and Greenway, S.C. (2019). Current methods for the maturation of induced pluripotent stem cell-derived cardiomyocytes. World J. Stem Cells *11*, 33–43.

Maddah, M., Heidmann, J.D., Mandegar, M.A., Walker, C.D., Bolouki, S., Conklin, B.R., and Loewke, K.E. (2015). A non-invasive platform for functional characterization of stem-cell-derived

cardiomyocytes with applications in cardiotoxicity testing. Stem Cell Reports *4*, 621–631.

Morris, T.A., Naik, J., Fibben, K.S., Kong, X., Kiyono, T., Yokomori, K., and Grosberg, A. (2020). Striated myocyte structural integrity: automated analysis of sarcomeric z-discs. PLoS Comput. Biol. *16*, e1007676.

Ostojic, A., Vrhovac, R., and Verstovsek, S. (2012). Ruxolitinib for the treatment of myelofibrosis: its clinical potential. Ther. Clin. Risk Manag. *8*, 95–103.

Parikh, S.S., Blackwell, D.J., Gomez-Hurtado, N., Frisk, M., Wang, L., Kim, K., Dahl, C.P., Fiane, A., Tonnessen, T., Kryshtal, D.O., et al. (2017). Thyroid and glucocorticoid hormones promote functional t-tubule development in human-induced pluripotent stem cell-derived cardiomyocytes. Circ. Res. *121*, 1323–1330.

Pioner, J.M., Santini, L., Palandri, C., Martella, D., Lupi, F., Langione, M., Querceto, S., Grandinetti, B., Balducci, V., Benzoni, P., et al. (2019). Optical investigation of action potential and calcium handling maturation of hiPSC-cardiomyocytes on biomimetic substrates. Int. J. Mol. Sci. *20*, 3799–3816. https://doi.org/10.3390/ijms20153799.

Putz, E.M., Majoros, A., Gotthardt, D., Prchal-Murphy, M., Zebedin-Brandl, E.M., Fux, D.A., Schlattl, A., Schreiber, R.D., Carotta, S., Muller, M., et al. (2016). Novel non-canonical role of STAT1 in natural killer cell cytotoxicity. Oncoimmunology *5*, e1186314.

Ruan, J.L., Tulloch, N.L., Razumova, M.V., Saiget, M., Muskheli, V., Pabon, L., Reinecke, H., Regnier, M., and Murry, C.E. (2016). Mechanical stress conditioning and electrical stimulation promote contractility and force maturation of induced pluripotent stem cell-derived human cardiac tissue. Circulation *134*, 1557–1567.

Rysa, J., Tokola, H., and Ruskoaho, H. (2018). Mechanical stretch induced transcriptomic profiles in cardiac myocytes. Sci. Rep. *8*, 4733.

Spletter, M.L., Barz, C., Yeroslaviz, A., Zhang, X., Lemke, S.B., Bonnard, A., Brunner, E., Cardone, G., Basler, K., Habermann, B.H., and Schnorrer, F. (2018). A transcriptomics resource reveals a transcriptional transition during ordered sarcomere morphogenesis in flight muscle. eLife *7*, 34058–34092. https://doi.org/10.7554/eLife.34058.001.

Sun, L., Ma, K., Wang, H., Xiao, F., Gao, Y., Zhang, W., Wang, K., Gao, X., Ip, N., and Wu, Z. (2007). JAK1-STAT1-STAT3, a key pathway promoting proliferation and preventing premature differentiation of myoblasts. J. Cell Biol. *179*, 129–138.

Takeda, M., Miyagawa, S., Fukushima, S., Saito, A., Ito, E., Harada, A., Matsuura, R., Iseoka, H., Sougawa, N., Mochizuki-Oda, N., et al. (2018). Development of in vitro drug-induced cardiotoxicity assay by using three-dimensional cardiac tissues derived from human induced pluripotent stem cells. Tissue Eng. Part C Methods *24*, 56–67.

Trenerry, M.K., Della Gatta, P.A., and Cameron-Smith, D. (2011). JAK/STAT signaling and human in vitro myogenesis. BMC Physiol. *11*, 6.

Ulmer, B.M., Stoehr, A., Schulze, M.L., Patel, S., Gucek, M., Mannhardt, I., Funcke, S., Murphy, E., Eschenhagen, T., and Hansen, A. (2018). Contractile work contributes to maturation of energy metabolism in hiPSC-derived cardiomyocytes. Stem Cell Reports *10*, 834–847.

Uosaki, H., Cahan, P., Lee, D.I., Wang, S., Miyamoto, M., Fernandez, L., Kass, D.A., and Kwon, C. (2015). Transcriptional landscape of cardiomyocyte maturation. Cell Rep. *13*, 1705–1716.

Varzideh, F., Pahlavan, S., Ansari, H., Halvaei, M., Kostin, S., Feiz, M.S., Latifi, H., Aghdami, N., Braun, T., and Baharvand, H. (2019). Human cardiomyocytes undergo enhanced maturation in embryonic stem cell-derived organoid transplants. Biomaterials *192*, 537–550.

Yang, X., Pabon, L., and Murry, C.E. (2014). Engineering adolescence: maturation of human pluripotent stem cell-derived cardiomyocytes. Circ. Res. *114*, 511–523.

Yang, X., and Papoian, T. (2018). Moving beyond the comprehensive in vitro proarrhythmia assay: use of human-induced pluripotent stem cell-derived cardiomyocytes to assess contractile effects associated with drug-induced structural cardiotoxicity. J. Appl. Toxicol. *38*, 1166–1176.

Yang, X., Rodriguez, M.L., Leonard, A., Sun, L., Fischer, K.A., Wang, Y., Ritterhoff, J., Zhao, L., Kolwicz, S.C., Jr., Pabon, L., et al. (2019). Fatty acids enhance the maturation of cardiomyocytes derived from human pluripotent stem cells. Stem Cell Reports *13*, 657–668.

Yoshida, S., Miyagawa, S., Fukushima, S., Kawamura, T., Kashiyama, N., Ohashi, F., Toyofuku, T., Toda, K., and Sawa, Y. (2018). Maturation of human induced pluripotent stem cell-derived cardiomyocytes by soluble factors from human mesenchymal stem cells. Mol. Ther. *26*, 2681–2695.

Stem Cell Reports, Volume *16*

Supplemental Information

Upregulation of the JAK-STAT pathway promotes maturation of human embryonic stem cell-derived cardiomyocytes

Beatrice Xuan Ho, Hongbing Yu, Jeremy Kah Sheng Pang, Jin-Hui Hor, Lee Chuen Liew, Piotr Szyniarowski, Christina Ying Yan Lim, Omer An, Henry He Yang, Colin L. Stewart, Woon Khiong Chan, Shi-Yan Ng, and Boon-Seng Soh

Up-regulation of JAK-STAT pathway promotes maturation of human embryonic stem cell-derived cardiomyocytes

Beatrice Xuan Ho[1,2,*], Hongbing Yu[1,*], Jeremy Kah Sheng Pang[1,2], Jin-Hui Hor[1,2,3], Lee Chuen Liew[1], Piotr Szyniarowski[4], Christina Ying Yan Lim[1], Omer An[5], Henry He Yang[5], Colin L. Stewart[4], Woon Khiong Chan[2], Shi Yan Ng[3,6,7,#] and Boon Seng Soh[1,2,#]

1 Disease Modeling and Therapeutics Laboratory, A*STAR Institute of Molecular and Cell Biology, 61 Biopolis Drive Proteos, Singapore 138673, Singapore.

2 Department of Biological Sciences, National University of Singapore, 117543, Singapore.

3 Neurotherapeutics Laboratory, A*STAR Institute of Molecular and Cell Biology, 61 Biopolis Drive Proteos, Singapore 138673, Singapore.

4 Institute of Medical Biology, 8A Biomedical Grove, Singapore 138648, Singapore

5 Cancer Science Institute of Singapore, National University of Singapore, Singapore 117599, Singapore

6 Department of Physiology, National University of Singapore, 2 Medical Dr, Singapore 117593, Singapore.

7 National Neuroscience Institute, Singapore 308433

* Equal contribution

\# Correspondence: **Shi-Yan Ng, Ph.D**

A*STAR Institute of Molecular and Cell Biology,

61 Biopolis Drive, Proteos, Singapore 138673.

Tel: (65) 6586 9525

Fax: (65) 6779 1117

Email: syng@imcb.a-star.edu.sg

Boon-Seng Soh, Ph.D

A*STAR Institute of Molecular and Cell Biology,

61 Biopolis Drive, Proteos, Singapore 138673.

Tel: (65) 6586 9530

Fax: (65) 6779 1117

Email: bssoh@imcb.a-star.edu.sg

Supplementary Information

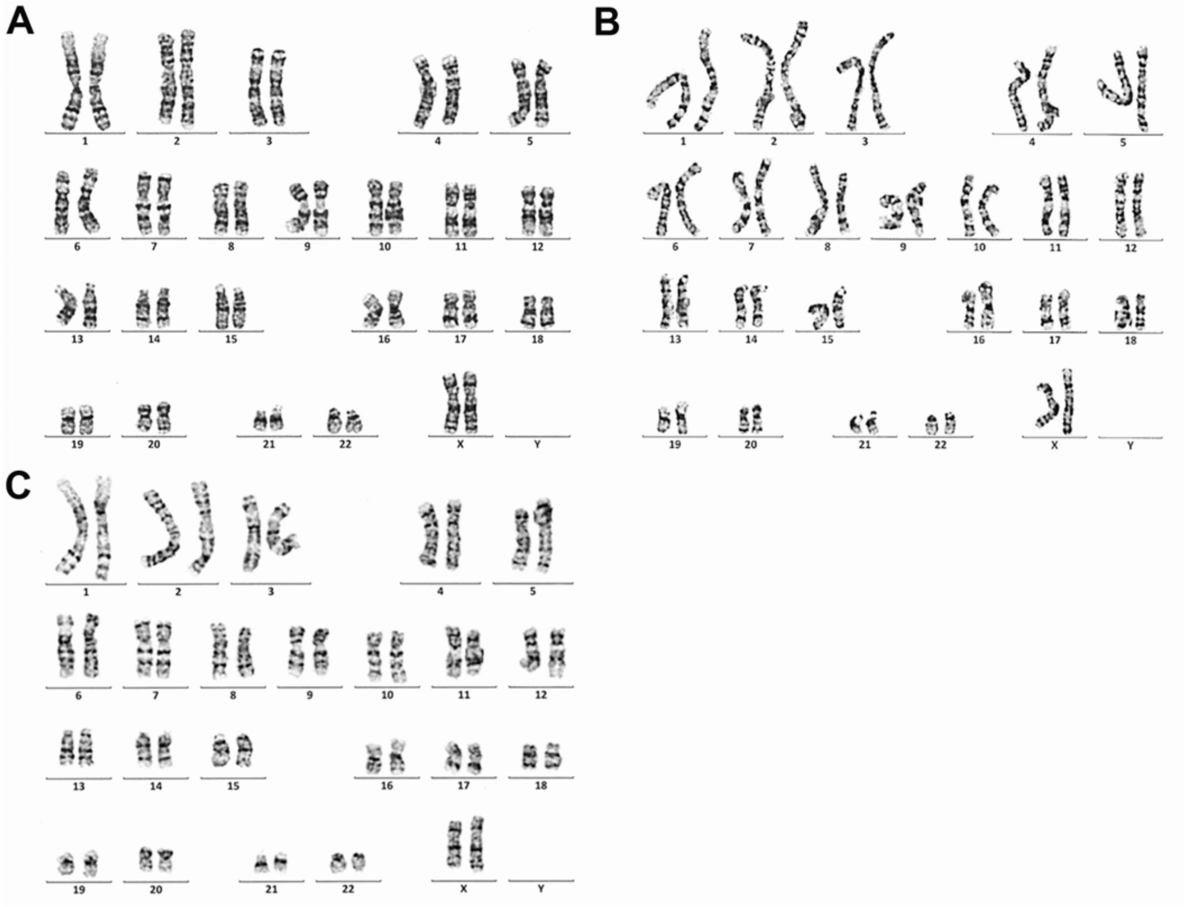

Figure S1. Karyotype of H7 HK1-GFP cell line.
(A-C) Normal female karyotype of human embryonic stem cell line, H7-HK1-GFP clones 1, 2 and 3 were used in this study.

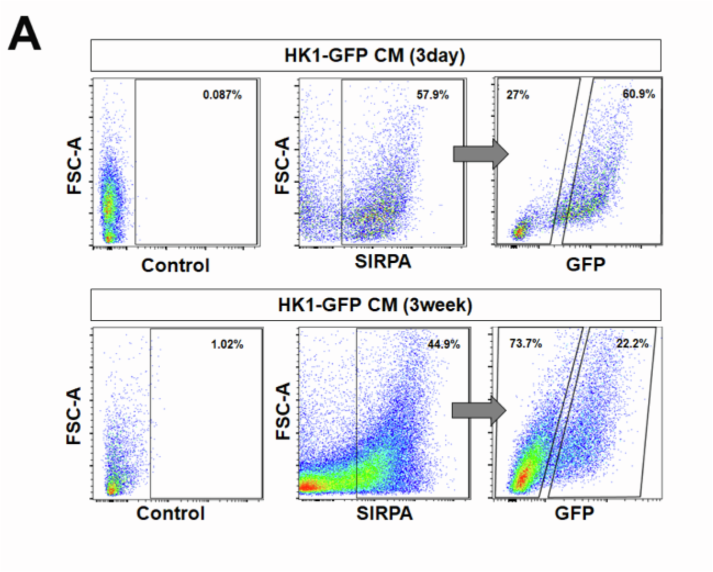

Figure S2. Representative flow cytometry analysis of H7 HK1-GFP derived CMs stained with SIRPA and sorted on 3-day and 3-week post-initial contraction.
(A) H7 HK1 derived CM, 3-day post initial contraction population showed 57.9% of the cells were SIRPA+, with 27% low-GFP and 60.9% high-GFP expressing CM. While H7 HK1 derived CM, 3-week post initial contraction population showed 44.9% of the cells were SIRPA+, with 73.7% low-GFP and 22.2% high-GFP expressing CM.

A

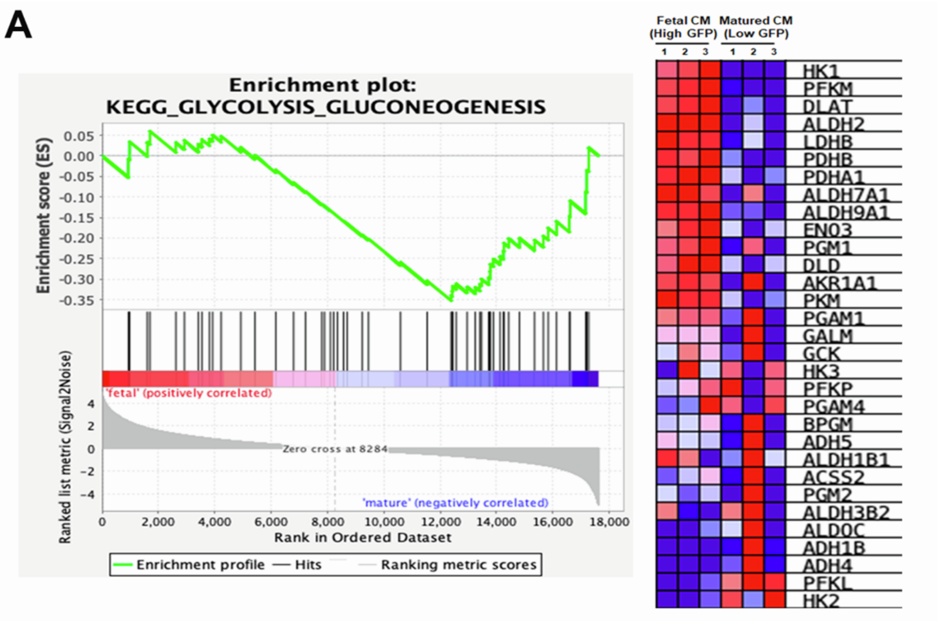

Figure S3. Heatmap showing relative expression of glycolysis and gluconeogenesis related genes.
(A) Gene set enrichment analysis (GSEA) showing downregulation of glycolysis and gluconeogenesis related genes in 3-week post initial contracting CM (expressing low GFP) compared to 3-day post initial contracting CM (expressing high GFP).

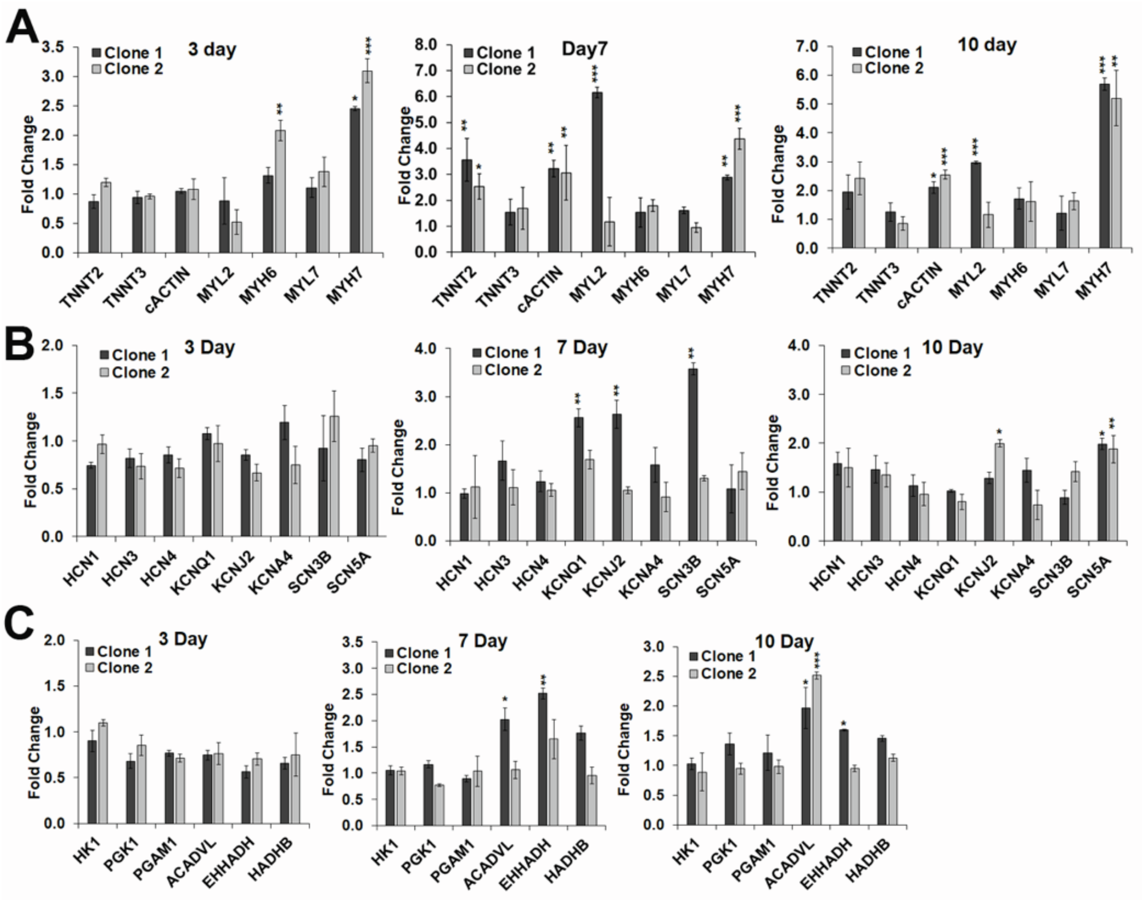

Figure S4. Relative mRNA expression levels of sarcomere, ion channel and metabolic genes in ES03-derived cardiomyocytes.

(A) Quantitative-PCR analysis illustrated increased mRNA transcript expression of sarcomere,

(B) ion channel, and

(C) metabolic genes, in IFN-γ-treated ES03-derived CM (clones 1 and 2), as compared to control. Data is represented as fold-change normalized to control samples, while expression of each gene was normalised to *β-ACTIN*. Data are presented as mean ± SD, n = 3 batches of independently differentiated groups. Data information: Statistical analysis was performed using students two-tailed T-test. * $P < 0.05$, ** $P < 0.01$, *** $P < 0.001$.

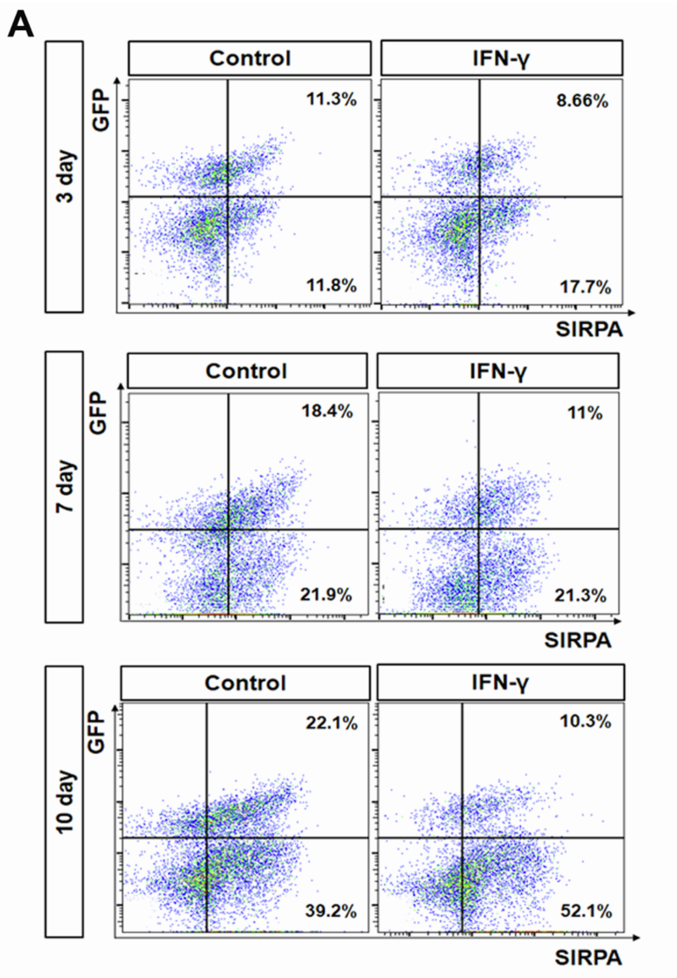

Figure S5. Flow cytometry analysis of relative HK1-GFP expression in H7-HK1 derived cardiomyocytes treated with IFN-γ.
(A) Representative flow cytometric analysis showing percentage of low:high GFP expressing cardiomyocytes in 3 day, 7 day and 10 day post IFN-γ-treated H7-derived CMs compared to control. n = 3 independently differentiated groups.

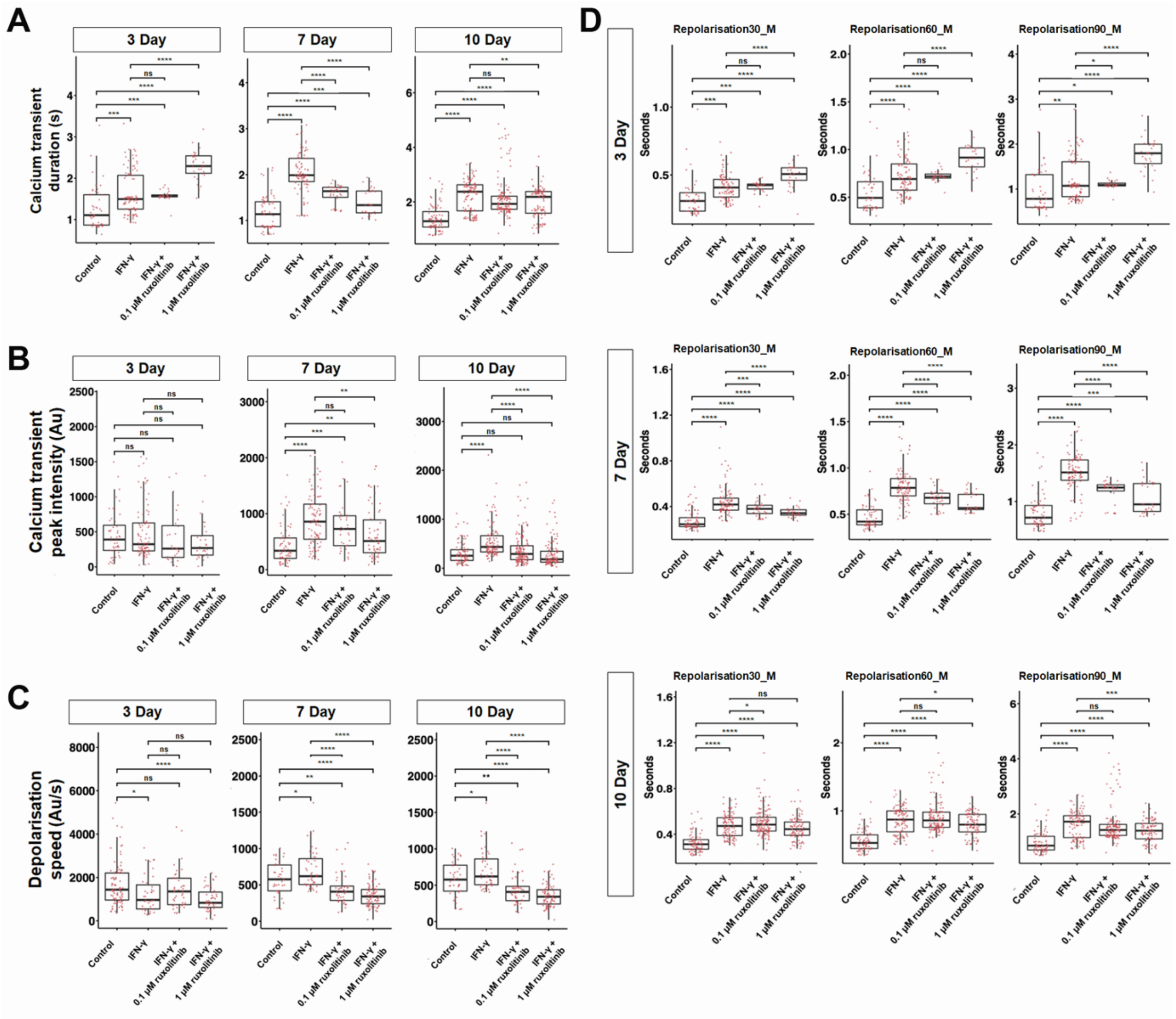

Figure S6. IFN-γ treated ES03-GCaMP6s expressing cardiomyocytes exhibit functional maturation. (A-D) Representative data of one of the two ES03-GCaMP6s clone derived CMs that were differentiated and analysed.

(A) Dot plot showing mean calcium transient duration (s),

(B) calcium transient peak intensity (Au),

(C) depolarisation speed (Au/s), and

(D) repolarisation duration (30th, 60th, 90th percent) per cell, measured across 30 seconds. n = 50 cells, 3 independently differentiated groups, 2 clones per cell line (ES03). Data information: Statistical analysis was performed using students two-tailed T-test. * $P < 0.05$, **$P < 0.01$, ***$P < 0.001$, ****$P < 0.0001$.

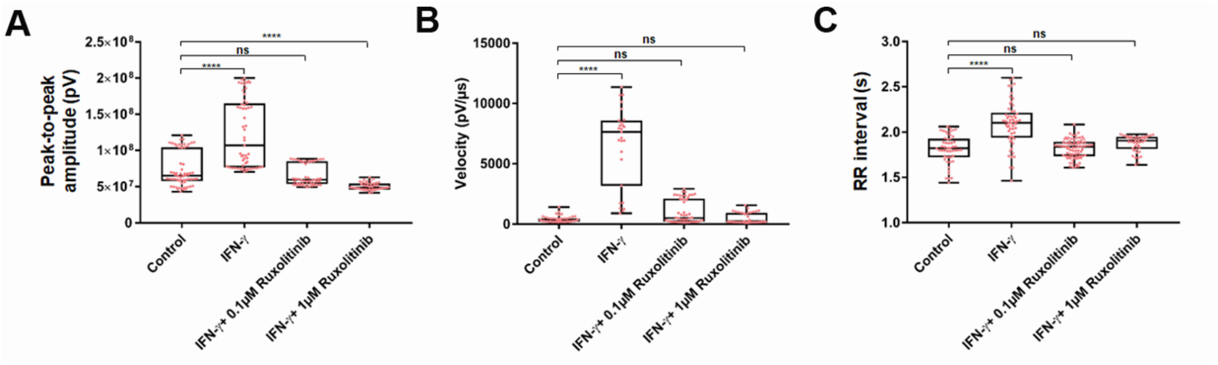

Figure S7. Electrophysiological assessment of IFN-γ-treated G608G human iPS-derived fetal cardiomyocytes using high-throughput multielectrode array (MEA) system.
(A) Dot plot showing mean peak-to-peak amplitude (pV),
(B) velocity (pV/ms), and
(C) RR interval of G608G human iPS-derived fetal CMs (10 days post IFN-γ treatment) measured across 5 min.
n = 3 independently differentiated groups. *$P < 0.05$, **$P < 0.01$, ***$P < 0.001$, ****$P < 0.0001$.

SUPPLEMENTAL MULTIMEDIA FILES

Video S1
Representative video of spontaneously contracting ES03-derived cardiomyocytes cultured in RPMI/B27.

Video S2
Representative video of spontaneously contracting ES03-derived cardiomyocytes cultured in RPMI/B27 supplemented with 25ng/ml IFN-γ.

SUPPLEMENTAL TABLES

Table S1
PCR primers used for genotyping of HK1-GFP report cell line.

**GT-LH-F**	CCATGTCAGCCCCTAACATCC
**GT-LH-R**	CGACGTCACCGCATGTTAGC
**GT-RH-F**	GCATCGCATTGTCTGAGTAGG
**GT-RH-R**	CCAGCCATTAAGCATCACTCG

Table S2
List of Quantitative-PCR primers used in this study.

Gene	Forward primer (5' – 3')	Reverse primer (5' – 3')
MEF2C	ATGGATGAACGTAACAGACAGGT	CGGCTCGTTGTACTCCGTG
NKX2.5	CCAAGGACCCTAGAGCCGAA	ATAGGCGGGGTAGGCGTTAT
TBX5	TTGCATGTATGCCAGCTCTG	CTGGTAGGGTAGCCTGTCC
cACTIN	TCCTGATGCGCATTTTTATTC	AACACCACTGCTCTAGCCACG
TNNT2	CAGAGCGGAAAAGTGGGAAGA	TCGTTGATCCTGTTTCGGAGA
TNNT3	GCCCACCTCAAGCAGGTG	TTGCGCCAGTCTCCCACCTC
HCN1	TGAAGCTGACAGATGGCTCTT	CTGGCAGTACGACGTCCTTT
HCN2	CTGATCCGCTACATCCATCA	AGATTGCAGATCCTCATCACC
HCN4	TCGACTCGGAGGTCTACAAG	GGTCGTAGGTCATGTGGAAG
KCNQ1	ATGGTGCGCATCAAGGAG	GATGAACAGTGAGGGCTTCC
KCNJ2	TGGCCAGGCTCATGTGTAG	CAAAAGGAAAAGCCCAGAAA
SCN3B	CATTCTGTAGCCCAGACGGG	GGGTAAGCTCAGCTCGGAAG
SCN5A	CACGCGTTCACTTTCCTTC	AAGAGCCGACAAATTGCCTA
CACNA1G	GAAGCTGATGGACGAGCTG	CCATCTCAGCTAGAGGGATCTG
MYL2	GCGGAGTGTGGAATTCTTCT	GTCAATGAAGCCATCCCTGT
HK1	TTCACGGAGCTGAAGGATGA	CTTCCTGAAGCGAGTCATGA
PGK1	GAATCACCGACCTCTCTCCC	GGGACAGCAGCCTTAATCCT
PGAM1	GAGCCCGACCATCCTTTCTA	CAGTACACGTTTCCCCTCCT
PPARA	GCTATCATTACGGAGTCCACG	TCGCACTTGTCATACACCAG
PPARG	GAGCCCAAGTTTGAGTTTGC	GCAGGTTGTCTTGAATGTCTTC
ACADVL	ATGGTGGTGGTTCTCTCGAG	GGCCTTGGAGATGCTTTTGA
EHHADH	GCATCGTGGAAAACAGCATC	CCGAGTCTACAGCAATCACAG
HADHB	ACCATGGCTTGTATCTCTGC	CTTGAGTGACGAATAGGGACATC
STAT1	ATGGCAGTCTGGCGGCTGAATT	CCAAACCAGGCTGGCACAATTG
IRF1	AAAGTCGAAGTCCAGCCGAG	TGTTGTAGCTGGAGTCAGGG
IRF2	AGGACAGTCCCATCTGGACA	ATTCCTCTTCCGCCAGTGTG
ISG20	ACACGTCCACTGACAGGCTGTT	ATCTTCCACCGAGCTGTGTCCA

β-ACTIN	CCAACCGCGAGAAGATGA	CCAGAGGCGTACAGGGATAG

Table S3
List of differentially expressed transcription factors of top 8 pathways between 3-week (relatively more matured CM expressing low GFP) and 3-day (fetal-like CM expressing high GFP) post initial contracting CMs (log2 fold change). Arranged in descending order from left (top to bottom), followed by right (top to bottom).

**Up-regulated transcription factors in matured low-GFP expressing CM**	**Down-regulated transcription factors in matured low-GFP expressing CM**
**Gene**	**Gene**
SP100	*TOP3A*
IRF8	*NUP153*
ATF3	*PIAS4*
IRF5	*CSNK1E*
IRF4	*PSMD10*
DDIT3	*CSNK2A2*
IRF1	*PRKACA*
IRF6	*NEK9*
ISG20	*FBXO5*
FRK	*VRK1*
CREB3L3	*MAPK3*
IRF9	*MDC1*
DNAJB9	*CHEK1*
EIF2AK3	*BRCA1*
STAT1	*CDC7*
NFKB1	*PLK4*
XBP1	*HERC2*
DNAJB11	*BUB1*
DNAJC3	*NEK2*
RNASEL	*PLK1*
ERN1	*PKMYT1*
IRF2	*AURKB*
PRKCD	*CDK1*
ATF4	*PRKCB*

Table S4
List of up-regulated genes 3-week (relatively more matured CM expressing low GFP) and 3-day (fetal-like CM expressing high GFP) post initial contracting CMs. All the genes were found to be associated with respective pathways with entities FDR less than 0.05 and P-value less than 0.01 listed.

Pathway name	Gene		P-value
**Neutrophil Degranulation**	*NEU1*	*LAMTOR3*	2.31E-08
	AHSG	*SERPINB6*	
	C3	*CD63*	
	HSPA6	*ORM2*	
	ANXA2	*DDX3X*	
	CTSH	*ITGAM*	
	FTL	*LAMTOR3*	
	SERPINA3	*SERPINB6*	
	CEACAM1	*SLPI*	
	GLIPR1	*HSPA1B*	
	LGALS3	*DNAJC3*	
	CTSS	*SLC2A5*	
	CD300A	*HSP90AA1*	
	FUCA1	*PDXK*	
	CXCL1	*LYZ*	
	AGPAT2	*OLFM4*	
	ORM1	*PRSS3*	
	HPSE	*RNASET2*	
	LRRC7	*ACAA1*	
	TLR2	*NFASC*	
	LPCAT1	*ASAH1*	
	SNAP25	*PRDX4*	
	QSOX1	*ABCA13*	
	ERP44	*C3AR1*	
	CD59	*CTSA*	
	DPP7	*STK11IP*	

	STBD1	*CD44*	
	MANBA	*AMPD3*	
	HEXB	*VNN1*	
	SLC15A4	*IDH1*	
	BRI3	*CD58*	
	CEACAM6	*ORMDL3*	
	ITGAL	*ARL8A*	
	AGA	*SERPINB1*	
	FUGA2	*TOLLIP*	
	LAMP2	*CD55*	
	FAF2	*ITGAX*	
	SVIP	*PYCARD*	
	TMEM173	*B2M*	
	ADAM8	*RAB18*	
	OLR1	*CD47*	
	RAB24	*PTAFR*	
	FCGR2A	*QPCT*	
	CYSTM1	*FTH1*	
	GSN	*ALDH3B1*	
	LRG1	*ALAD*	
	TMBIM1	*CYBA*	
	P2RX1	*A1BG*	
	PLD1	*NIT2*	
	HSPA1A	*ARHGAP45*	
	LRMP	*CTSZ*	
	FRK	*COPB1*	
	MAGT1	*HLA-C*	
	S100P	*RAP1A*	
	NBEAL2	*DSC1*	

	CHI3L1	*ANO6*	
	CD68	*VAMP8*	
	NFKB1	*ALOX5*	
	CD63	*HLA-B*	
	ORM2	*CPPED1*	
	DDX3X	*ITGAV*	
	ITGAM	*PRKCD*	
	SNAP23	*MMP9*	
	NHLRC3	*TNFAIP6*	
**Interferon alpha/beta signaling**	*IRF1*	*MX2*	7.19E-08
	GBP2	*IFNAR1*	
	PSMB8	*XAF1*	
	MX1	*IFNAR2*	
	STAT1	*IRF9*	
	IRF5	*IRF6*	
	ISG20	*HLA-F*	
	IFIT2	*RNASEL*	
	IRF2	*OAS3*	
	IRF8	*IP6K2*	
	OAS1	*HLA-C*	
	IFIT3	*TYK2*	
	JAK1	*RSAD2*	
	HLA-G	*HLA-A*	
	IRF4	*HLA-B*	
	ATF3	*EIF2AK3*	
	HERPUD1	*ASNS*	
	DDIT3	*TSPYL2*	
	CREBRF	*CREB3L3*	
	CXCL8	*SEC31A*	

**Unfolded protein response (UPR)**	*XBP1*	*KDELR3*	9.90E-08
	SRPRB	*DNAJC3*	
	CXXC1	*HSPA5*	
	DNAJB9	*SSR1*	
	CCL2	*SRPRA*	
	IGFBP1	*ACADVL*	
	EDEM1	*DNAJB11*	
	PDIA5	*HSP90B1*	
	LMNA	*PPP2R5B*	
	WFS1	*MYDGF*	
	ATF4	*YIF1A*	
	GFPT1	*WIPI1*	
	SERP1	*ERN1*	
**Interferon gamma signaling**	*GBP1*	*HLA-G*	9.90E-08
	IRF1	*IRF4*	
	TRIM21	*PTPN2*	
	GBP2	*GBP7*	
	SP100	*IRF9*	
	STAT1	*IRF6*	
	IRF5	*HLA-F*	
	MT2A	*IFI30*	
	TRIM22	*CD44*	
	IRF2	*B2M*	
	IRF8	*OAS3*	
	ICAM1	*PTAFR*	
	GBP3	*HLA-C*	
	TRIM25	*HLA-A*	
	TRIM26	*HLA-B*	
	OAS1	*PRKCD*	

	JAK1	*TRIM29*	
	TRIM31	*IFNGR2*	

Table S5
List of down-regulated genes in 3-week (relatively more matured CM expressing low GFP) and 3-day (fetal-like CM expressing high GFP) post initial contracting CMs. All the genes were found to be associated with respective pathways with entities FDR less than 0.05 and P-value less than 0.01 listed.

Pathway name	Gene		P-value
Mitotic Prometaphase	*DYNC1I1*	*CENPN*	4.57E-17
	SMC1A	*CENPF*	
	ZWILCH	*CENPE*	
	ZWINT	*PRKAR2B*	
	TUBA1B	*BUB1*	
	SPC25	*KIF2C*	
	ERCC6L	*CLASP2*	
	CDCA5	*NDE1*	
	DYNLL2	*HAUS5*	
	CSNK2A2	*CENPL*	
	NUP107	*NDC80*	
	NCAPG	*AURKB*	
	CENPO	*HAUS1*	
	MAD1L1	*NCAPD2*	
	CENPU	*PLK4*	
	BUB1B	*CENPH*	
	SMC4	*SMC2*	
	CENPI	*MAPRE1*	
	PLK1	*NINL*	
	KNL1	*NUF2*	
	SGO1	*MZT1*	
	CEP72	*CDC20*	
	CENPK	*MAD2L1*	
	DCTN1	*NEK2*	
	INCENP	*NUMA1*	

	PPP2R5D	*NEDD1*	
	SGO2	*KNTC1*	
	BIRC5	*PRKACA*	
	SKA1	*PPP1CC*	
	TUBB	*CENPA*	
	SPDL1	*SPC24*	
	YWHAE	*HAUS4*	
	CKAP5	*CCNB2*	
	NCAPH	*CCNB1*	
	XPO1	*CSNK1E*	
	CENPM	*ALMS1*	
	CDCA8	*CDK1*	
**Cell Cycle checkpoints**	*HERC2*	*SGO2*	**1.40E-16**
	DYNC1I1	*BIRC5*	
	MDC1	*SKA1*	
	PKMYT1	*CCNE1*	
	ZWILCH	*SPDL1*	
	ZWINT	*YWHAE*	
	CLSPN	*CKAP5*	
	RMI2	*XPO1*	
	MCM5	*CENPM*	
	MCM2	*CDCA8*	
	SPC25	*CDK1*	
	TP53BP1	*DNA2*	
	ERCC6L	*CENPN*	
	CDC45	*CENPF*	
	MCM4	*CENPE*	
	MCM6	*BUB1*	
	EXO1	*KIF2C*	

	CDC25A	*CLASP2*	
	CDC7	*NDE1*	
	MCM3	*PSMD10*	
	DYNLL2	*CENPL*	
	MCM8	*NDC80*	
	GTSE1	*AURKB*	
	MCM10	*UBE2C*	
	BRCA1	*CCNA2*	
	H2AFX	*CENPH*	
	CDC6	*MCM7*	
	RFC3	*TOP3A*	
	CDKN1B	*PIAS4*	
	NUP107	*ATRIP*	
	CENPO	*MAPRE1*	
	CHEK1	*NUF2*	
	MAD1L1	*CDC20*	
	CENPU	*MAD2L1*	
	BUB1B	*RMI1*	
	CENPI	*KNTC1*	
	PLK1	*RPA2*	
	KNL1	*PSMB1*	
	SGO1	*RFC4*	
	PHF20	*RFC2*	
	CENPK	*ANAPC1*	
	BLM	*PPP1CC*	
	CDC23	*CENPA*	
	INCENP	*ORC6*	
	PPP2R5D	*SPC24*	
	PSME3	*CDC25C*	

	CCNB1	*CCNB2*	
**Amplification of signal from the kinetochores**	*DYNC1I1*	*BIRC5*	**2.67E-15**
	ZWILCH	*SKA1*	
	ZWINT	*SPDL1*	
	SPC25	*CKAP5*	
	ERCC6L	*XPO1*	
	DYNLL2	*CENPM*	
	NUP107	*CDCA8*	
	CENPO	*CENPN*	
	MAD1L1	*CENPF*	
	CENPU	*CENPE*	
	BUB1B	*BUB1*	
	CENPI	*KIF2C*	
	PLK1	*CLASP2*	
	KNL1	*NDE1*	
	SGO1	*CENPL*	
	CENPK	*NDC80*	
	INCENP	*AURKB*	
	PPP2R5D	*CENPH*	
	SGO2	*MAPRE1*	
	NUF2	*KNTC1*	
	CDC20	*PPP1CC*	
	MAD2L1	*CENPA*	
	SPC24		
	DYNC1I1	*SKA1*	
	ZWILCH	*SPDL1*	
	ZWINT	*CKAP5*	
	SPC25	*XPO1*	
	ERCC6L	*CENPM*	

**Mitotic Spindle Checkpoint**	*DYNLL2*	*CDCA8*	**2.39E-14**
	NUP107	*CENPN*	
	CENPO	*CENPF*	
	MAD1L1	*CENPE*	
	CENPU	*BUB1*	
	BUB1B	*KIF2C*	
	CENPI	*CLASP2*	
	PLK1	*NDE1*	
	KNL1	*CENPL*	
	SGO1	*NDC80*	
	CENPK	*AURKB*	
	CDC23	*UBE2C*	
	INCENP	*CENPH*	
	PPP2R5D	*MAPRE1*	
	SGO2	*NUF2*	
	BIRC5	*CDC20*	
	MAD2L1	*PPP1CC*	
	KNTC1	*CENPA*	
	ANAPC1	*SPC24*	

SUPPLEMENTAL EXPERIMENTAL PROCEDURES

Generation of clonal H7 HK1-EGFP reporter cell line

sgRNA sequences were identified using the online CRISPR design tool (crispr.mit.edu). In brief, 100 base pair DNA sequences of the human HK1 gene flanking the stop codon was used for designing the sgRNAs. Two pairs of sgRNAs were selected for cloning into PX459 Cas9 vector. For construction of the HK1 donor vector, V5-2A-EGFP sequence was inserted into pUC19 vector. The HK1 left homologue arm without stop code was inserted upstream of the V5-2A-EGFP sequence, while the HK1 right homologue arm was inserted downstream of the gene. Both left and right homologue arms were validated through sequencing. 10ug HK1 sgRNA and 10ug HK1 donor vector were electroporated into $1x10^6$ the human H7 hES cell line (Thermos-fisher Neon 10ul kit, 1400 V, 30 sec). After 1 week, the electroporated human ES cells were sorted twice for EGFP expression. Single H7 HK1-EGFP positive cells were seeded into 96-well plate for clonal expansion.

PCR validation of HK1 reporter clones

Briefly, GoTaq® Flexi DNA Polymerase (Promega, U.S.A., M829) kit was used in accordance to manufacturer's recommendations. The PCR primers used for genotyping as shown in Table S1.

hESC-derived cardiomyocyte differentiation, sample collection for RNA sequencing and IFN-γ treatment

Upon 80-90% confluence, H7, ES03 and G608G hES/iPS cells were differentiated into CMs via Wnt signalling modulation method as described by Lian *et al*., 2013 (Lian et al. 2013). CMs were FAC sorted after 3-days post intial contraction (expressing high HK1-GFP) and 3-weeks post intial contraction (expressing low HK1-GFP) for RNA sequencing (Refer to Figure 2A).

Prior to IFN-γ treatment, hES-derived CMs were dissociated with accutase and purified with human PSC-derived cardiomyocyte isolation kit (Miltenyi Biotec, Germany) as described on manufacturer's instructions. Enriched cardiomyocyte population was seeded using RPMI/B27 with 5 μM of Y27632 (Miltenyi Biotec, Germany) onto on culture plates coated with Matrigel® matrix (Corning, U.S.A.) diluted in DMEM/F12 (Gibco, U.S.A.). On day 10, contracting CMs were treated with 25ng/ml IFN-γ (Miltenyi Biotec; 130-096-482) and ruxolitinib (Selleckchem; NCB018424) in RPMI/B27 for 3 days, 7 days and 10 days, as illustrated in figure 4A.

SDS-PAGE and western blot

Cells were lysed with RIPA buffer (ThermoScientific, U.S.A.) with the addition of cOmplete™ Protease

Inhibitor Cocktail (Sigma Aldrich, U.S.A.). Cells that require preservation of phosphorylation state were lysed with PhosphoSafe™ Extraction Reagent (Novagen; #71296-3). Protein extracts were then quantified using Pierce™ BCA Protein Assay Kit (Thermo Scientific, U.S.A.) in accordance to manufacturer's recommendations. Protein lysates were resolved in 8-12% SDS-PAGE gels in Tris-Glycine-SDS buffer, transferred to a PDVF membrane (Bio-Rad, U.S.A.) using the Trans-Blot® Turbo™ transfer system (Bio-Rad, U.S.A.) or conventional wet transfer methods. Primary antibodies used include mouse anti-V5 antibody (ThermoScientific, U.S.A.), mouse anti-HK1 antibody (ThermoScientific, U.S.A.), mouse anti α-tubulin (Cell signaling technologies, #2148S), mouse anti-β-actin (Cell signaling technologies, 8H10D10), rabbit anti-GAPDH (Cell signaling technologies, D16H11), rabbit anti-STAT1 (Cell signaling technologies, D1K9Y), rabbit anti-phospho STAT1 (Cell signaling technologies, Y701), rabbit anti-MYH7 (Abcam, ab172967) and anti-MYH7 (Santa cruz, sc-53090) were prepared according to manufacturer's instructions. Secondary antibodies conjugated with horseradish peroxidase (Santa Cruz Biotechnology, U.S.A.) were used. Membranes were then exposed to Clarity™ ECL Western Substrate (Bio-rad, U.S.A.).

Fluorescence-activated cell sorting and flow cytometric analysis

Cardiomyocyte were first dissociated using StemPro-Accutase®(Gibco, U.S.A.) and stained with SIRPA antibody conjugated with SIRPA-conjugated PE-Cy7 (NEB) (1:250 dilution) in blocking buffer (1% FBS in PBS) for 2 h at 37 °C and washed thoroughly with PBS before the cells were resuspended in fluorescence-activated cell sorting (FACs) buffer (0.5% FBS and 1% BSA in PBS). FACS was performed using the LSR II (BD Biosciences, U.S.A.).

For intracellular staining, cells were fixed in 4% PFA (Nacalai Tesque, Japan) at room temperature for 10 min, before incubation in blocking buffer (PBS with 1% Fetal Bovine Serum; Gibco, U.S.A.) with 0.1% Triton-X (Promega, U.S.A.) for 10 min, followed by staining in blocking buffer with rabbit anti-MYH7 antibody (1:250, ab172967; Abcam) for 2 h at room temperature. The cells were washed with PBS and centrifuged at 1200rpm for 5min, the cells were stained in Alexa Fluor® 594 (1:1000) (Invitrogen, U.S.A.) for 1-1.5 h at room temperature in the dark. The cells were washed thoroughly with PBS before resuspension in FACS buffer (0.5% FBS, 1%BSA in PBS). Flow cytometry analysis was performed with LSR II (BD Biosciences, U.S.A.).

RNA Extraction and RT-qPCR

CMs were lysed in 300 µl of TRIzol reagent (Invitrogen, U.S.A.) followed by RNA extraction and precipitation from the aqueous phase and removal of genomic DNA. RNA samples (250-500 ng) were reverse transcribed using High-Capacity cDNA Reverse Transcription Kit (Applied Biosystems, U.S.A.). qPCR was performed using the FAST SYBR Green Mix (Applied Biosystems, U.S.A.), 0.5 µM of specific primers (Table S2) and ~5 ng of cDNA. $\Delta\Delta C_T$-based relative quantification method was adopted for qPCR analysis using the QuantStudio 5 384-well Block Real-Time PCR system (Applied Biosystems, U.S.A.). Data is presented as fold-change where CT values were normalised to *β-ACTIN*. Data presented are representative of three independent experiments with error bars indicative of the standard deviation (SD) unless otherwise stated.

Metabolic flux analysis using Seahorse XFe96 Analyzer

Extracellular acidification rates (ECAR) were measured using a XFe96 Seahorse Biosciences Extracellular Flux Analyzer (Agilent Technologies). Purified CMs were plated onto a Matrigel pre-coated Seahorse 96-well plate at 125,000 CMs per well 24 hours prior to the assay. Culture media were changed with 175ul of fresh Seahorse DMEM basal medium 45 minutes prior to the assay. Seahorse analyzer injection ports were filled with 10mM glucose, 1µM oligomycin and 50mM 2-DG, following the manufacturer's instructions. Levels of ECAR were recorded, normalized and quantified based on manufacturer's instructions.

Immunostaining, confocal microscopy and image analysis

Cells were then fixed using 4% PFA (Nacalai-Tesque, Japan) for 10 min at room temperature, followed by permeabilisation with 0.2% Triton-X (Promega, U.S.A.) for 10 min. The cells were then treated with blocking buffer for 30 min. Primary antibody anti-sarcomeric α-actinin (1:150, ab9465, Abcam) was incubated at 4 °C overnight, followed by incubation with the appropriate Alexa Fluor® 488 and 594 fluorescent dyes for 1.5 hours, and counter-stained with 4,6-diamindino-2-phenylindole (DAPI) (AAT Biorequest, U.S.A.). For this study, primary antibody used were mouse α-actinin (1:150, ab9465, Abcam). Imaging was performed using the Olympus Fluoview inverted confocal microscope (Olympus, U.S.A.) using oil immersion for 100x optical zoom. Sarcomere length and myofibril width were measured with freehand drawing tools in Fiji based on α-actinin staining. Sarcomere length and myofibril width were measured automatically using a custom Fiji plug-in, MyofibrilJ, available from (https://imagej.net/MyofibrilJ). All measurements were based on α-actinin staining. Full details of analysis was performed as mentioned by Spletter *et al.,* (Spletter et al. 2018). Data were recorded in Excel and statistical analysis comparing two groups were performed by mean of a two-tailed

unpaired Student's t-test. P values lower than 0.05 were considered significant. A computational tool, ZlineDetection was performed on MATLAB to determine skewness of continuous z-line (CZL) and z-line fraction as described by *Morris et al* (Morris et al. 2020). All measurements were based on α-actinin (green) and phalloidin (red) staining. Statistical analysis comparing two groups were performed by mean of a two-tailed unpaired Student's t-test. P values lower than 0.05 were considered significant.

Mitotracker staining, image acquisition and image analysis

MitoTracker® Red FM, far red-fluorescent dye (abs/em ~581/644 nm) was used to stain mitochondria in live CMs. Images of FAC sorted CMs (low/high-GFP expressing) on 96-well plates were acquired using the high content microscope Operetta (Perkin Elmer) using the 20x objective. Image analyses including intensity measurements were performed using Columbus (Perkin Elmer). Nuclei were detected in live cells with hoescht (blue) stain, with dead cells filtered based on abnormally high hoescht intensity and small ($<20\ \mu m^2$) nuclei area. Intensity of mitotracker staining within cytoplasm was determined and a cut-off above background intensity was used to identify CMs.

Gene expression profiling and pathway enrichment analysis

Gene expression profiling and pathway enrichment analysis have been performed by using CSI NGS Portal (An et al. 2019). Briefly, raw fastq files were trimmed by Trimmomatic (Bolger, Lohse, and Usadel 2014) for adapter removal. The clean reads were aligned to the reference human genome (hg19) by using STAR (Dobin et al. 2013) (v2.7.3a) with default parameters. The gene expression quantification was done by using HTSeq-count(Anders, Pyl, and Huber 2015) (v0.11.2) in strand-specific mode "-s reverse" to obtain raw read counts for each gene, and read counts only from the sense strand are used. First, PCA and hierarchical clustering were performed with regionReport (Collado-Torres, Jaffe, and Leek 2015) to compare the gene expression profiles of the samples, by using the top 500 genes with the highest variance across the samples. Then the differential gene expression analysis was performed by using DESeq2 (Love, Huber, and Anders 2014) (v1.24.0) starting from the raw read counts by comparing the samples from C9 Matured Low GFP to C9 Fetal Hi GFP after collapsing the three samples within each group as replicates. The genes that are not expressed or lowly expressed (read counts <= 2 on average per sample) were removed from the analysis. In total, 3128 and 2630 genes were significantly up- and down-regulated, respectively, in the 3-week post initial contracting CMs, as compared to the 3-day post initial contracting CMs, after correction for multiple hypothesis testing ($P_{adj} < 0.05$, using

Benjamini-Hochberg method). These differentially expressed genes were used for the pathway enrichment analysis (ReactomePA database) (Yu and He 2016). Additionally, gene set enrichment analysis (GSEA(Subramanian et al. 2005)) was performed by using the gene expression data for all the genes normalized by DESeq2 (Love, Huber, and Anders 2014). To be comprehensive, all pathways and gene sets were selected as the input for the enrichment analyses. The significantly altered pathways were identified and plotted as barplot by using R packages ReactomePA (Yu and He 2016) and enrichplot (Yu 2018), respectively.

Fluorescent imaging of intracellular calcium transient

Intracellular calcium kinetics was measured using H7- and ES03-GCaMP6s derived CMs. In brief, the cells were seeded on a 24 well plate pre-coated with matrigel. Prior to imaging, the cells were washed with PBS, and fresh RPMI/B27 medium was added. Spontaneous calcium transients of 3 day, 7 day, 10 day IFN-γ , 0.1μM and 1μM ruxolitinib treated CMs and untreated controls were imaged under a 20x objective Nikon ECLIPSE Ti-S fluorescent microscope and recorded using an Andor Zyla 4.2 sCMOS for up to 1 minute. Calcium transients based on fluorescence intensity over time were computed into contraction peaks in Rstudio. The contraction peaks were computed into the following variables; (1) calcium transient duration (seconds), (2) calcium transient peak intensity (Au), (3) depolarisation speed (mean fluorescence per second), and (4) repolarisation duration (Repolarisation30_M, Repolarisation60_M, Repolarisation90_M) by taking the mean of the contraction intervals respectively. Statistical significance using Student's *t*-test, two sided based on assumed normal distribution.

Multielectrode array (MEA) recording

PEDOT-coated 24-well micro-gold multielectrode array dishes (catalog number 24W300/30G-288, Multi Channel Systems MCS, Germany) contained an array of 12 electrodes per well. Fetal-like CMs (7-10 days) were seeded at a density of 350,000 cells/well that were pre-coated with Matrigel® matrix (Corning, U.S.A.) diluted in DMEM/F12 (Gibco, U.S.A.). CMs were treated with 25ng/ml IFN-γ, 0.1μM and 1μM Ruxolitinib in RPMI/B27 the following day. Field potentials (FP) were recorded 10 days after IFN-γ treatment, at a sampling rate of 20 kHz unless otherwise noted, with a recording duration of 5 min and 2 min wash-in phase. High- and low-pass filter cut-off frequency of 1 Hz and 3500 Hz, respectively were used for data acquisition. A separate software, Multiwell-Analyzer (Multi Channel Systems MCS, Germany) was used to analyse and extract data from the recording. The "RR Internal" measures the time span between two heartbeats, measured from the

detection time stamp of one QRS-component to the next. "Peak-to-Peak amplitude" measures the amplitude between the maximum and the minimum of a Na peak. “Velocity” was measured based on "Peak-to-Peak amplitude" divided by the duration.

Statistical analysis

Quantitative PCR (qPCR) values are expressed as mean ± SD (unless otherwise stated), n = 3 independently differentiated groups. Results were tested for statistical significance using Student’s *t*-test, two sided based on assumed normal distribution. * $P < 0.05$, **$P < 0.01$, ***$P < 0.001$.

References

An, Ö., K. Tan, Y. Li, J. Li, C. Wu, B. Zhang, L. Chen, and H. Yang. 2019. 'CSI NGS Portal: An Online Platform for Automated NGS Data Analysis and Sharing', *Preprints*, 2019100146.

Anders, S., P. T. Pyl, and W. Huber. 2015. 'HTSeq--a Python framework to work with high-throughput sequencing data', *Bioinformatics*, 31: 166-9.

Bolger, A. M., M. Lohse, and B. Usadel. 2014. 'Trimmomatic: a flexible trimmer for Illumina sequence data', *Bioinformatics*, 30: 2114-20.

Collado-Torres, L., A. E. Jaffe, and J. T. Leek. 2015. 'regionReport: Interactive reports for region-level and feature-level genomic analyses', *F1000Res*, 4: 105.

Dobin, A., C. A. Davis, F. Schlesinger, J. Drenkow, C. Zaleski, S. Jha, P. Batut, M. Chaisson, and T. R. Gingeras. 2013. 'STAR: ultrafast universal RNA-seq aligner', *Bioinformatics*, 29: 15-21.

Lian, X., J. Zhang, S. M. Azarin, K. Zhu, L. B. Hazeltine, X. Bao, C. Hsiao, T. J. Kamp, and S. P. Palecek. 2013. 'Directed cardiomyocyte differentiation from human pluripotent stem cells by modulating Wnt/beta-catenin signaling under fully defined conditions', *Nat Protoc*, 8: 162-75.

Love, M. I., W. Huber, and S. Anders. 2014. 'Moderated estimation of fold change and dispersion for RNA-seq data with DESeq2', *Genome Biol*, 15: 550.

Morris, T. A., J. Naik, K. S. Fibben, X. Kong, T. Kiyono, K. Yokomori, and A. Grosberg. 2020. 'Striated myocyte structural integrity: Automated analysis of sarcomeric z-discs', *PLoS Comput Biol*, 16: e1007676.

Spletter, M. L., C. Barz, A. Yeroslaviz, X. Zhang, S. B. Lemke, A. Bonnard, E. Brunner, G. Cardone, K. Basler, B. H. Habermann, and F. Schnorrer. 2018. 'A transcriptomics resource reveals a transcriptional transition during ordered sarcomere morphogenesis in flight muscle', *Elife*, 7.

Subramanian, A., P. Tamayo, V. K. Mootha, S. Mukherjee, B. L. Ebert, M. A. Gillette, A. Paulovich, S. L. Pomeroy, T. R. Golub, E. S. Lander, and J. P. Mesirov. 2005. 'Gene set enrichment analysis: a knowledge-based approach for interpreting genome-wide expression profiles', *Proc Natl Acad Sci U S A*, 102: 15545-50.

Yu, G., and Q. Y. He. 2016. 'ReactomePA: an R/Bioconductor package for reactome pathway analysis and visualization', *Mol Biosyst*, 12: 477-9.

Yu, Guangchuang. 2018. 'Visualization of Functional Enrichment Result', *R package version 1.2.0*.
